# The RNA binding protein CARHSP1 facilitates tumor growth, metastasis and immune escape by enhancing IL-17RA mRNA stabilization in prostate cancer

**DOI:** 10.1186/s13578-025-01371-4

**Published:** 2025-03-07

**Authors:** YiFan Jiang, Yanan Wang, KaiHua Xue, JianBin Ma, Shan Xu, Ke Wang, Peng Guo

**Affiliations:** 1 Department of Urology, The First Affiliated Hospital of Xi’an Jiaotong University, 277 Yan-ta West Road, Xi’an, 710061 Shaanxi China; 2 Key Laboratory for Tumor Precision Medicine of Shaanxi Province, Xi’an, 710061 Shaanxi China; 3 Oncology Research Lab, Key Laboratory of Environment and Genes Related to Diseases, Ministry of Education, Xi’an, 710061 Shaanxi China

**Keywords:** CARHSP1, Prostate cancer, IL-17RA, RNA binding protein, JAK-STAT3 signaling pathway, NF-κB signaling pathway, PD-L1

## Abstract

**Background:**

Calcium-regulated heat-stable protein 1 (CARHSP1) has been identified as a cold shock domain (CSD) protein family member, participating in the regulation of ribosomal translation, mRNA degradation, and the rate of transcription termination. However, there is an extremely limited understanding of the function of CARHSP1 as an RNA binding protein (RBP) in prostate cancer (PCa).

**Methods:**

The expression pattern of CARHSP1 and the correlation between the CARHSP1 expression and clinical prognosis in PCa patients were analyzed by using multiple public databases. In vitro and in vivo functional assays were conducted to assess the role of CARHSP1. The mechanisms of CARHSP1 function on IL-17RA were identified by RNA pull-down and RNA stability assays. A co-culture model of Jurkat cells and PCa cells was established to investigate the potential role of CARHSP1 in tumor immunity of PCa.

**Results:**

CARHSP1 was highly expressed in PCa, and correlated with advanced characteristics of PCa and unfavorable prognosis in PCa patients. Moreover, knockdown of CARHSP1 significantly dampened the capacity of proliferation, migration, invasion, and immune evasion of PCa cells in vitro and in vivo. Mechanistically, the RNA-binding protein CARHSP1 selectively bound to the mRNA of IL-17RA, resulting in the increased expression of both IL-17RA mRNA and protein. Downregulating expression of CARHSP1 shortened the half-life of IL-17RA mRNA and reduced its expression. Subsequently, the downstream pathways of IL-17RA, JAK-STAT3 signaling pathway and NF-κB signaling pathway, were activated by CARHSP1 and contributed to the malignant phenotype of PCa cells.

**Conclusions:**

In conclusion, our results demonstrated that the increased expression of CARHSP1 in PCa is correlated with advanced clinical characteristics and unfavorable prognosis, and CARHSP1 may promote the progression of PCa through enhancing the mRNA stability of IL-17RA and activating its downstream pathways. These results suggest that CARHSP1 is an important regulator of tumor microenvironment in PCa, and CARHSP1-IL-17RA axis could be potential novel therapeutic targets for PCa.

**Supplementary Information:**

The online version contains supplementary material available at 10.1186/s13578-025-01371-4.

## Introduction

Prostate cancer (PCa) is the second most common cancer and the fifth leading cause of cancer death in men worldwide [1]. In recent years, many therapeutic strategies for PCa have contributed to a reduction in mortality, such as endocrine therapy, radiotherapy, and surgical operation. Nevertheless, the foregoing approaches display limited efficacy, and one-third of PCa patients will recur, advance to castration-resistant PCa stage (CRPC), and develop distant organ metastases, resulting in shorter overall survival [2, 3]. Therefore, there is an urgent need to investigate the function of key molecules in the progression of PCa and explore more potential therapeutic targets to improve the survival prognosis of patients.

Calcium-regulated heat-stable protein 1 (CARHSP1), also known as CRHSP-24, is a cytoplasmic protein and exhibits a broad tissue distribution, which is comprised of 147 amino acids [4]. CARHSP1 has been identified as a cold shock domain (CSD) protein family member, participating in the regulation of ribosomal translation, mRNA degradation, and the rate of transcription termination [5]. Each CSD possess two nucleic acid-binding motifs, making CSD proteins possible to bind to polypyrimidine regions and regulate the stability of single-stranded RNA or DNA [6]. Recently, several studies have demonstrated that CARHSP1 binds to the AU-rich element (ARE) in the 3’-UTR of TNF-α mRNA to regulate the stability of TNF-α mRNA [7–9]. Actually, CARHSP1 was originally identified as a physiological substrate for the calcineurin [4], and the published literature of CARHSP1 focused primarily on the signaling pathways that phosphorylate and dephosphorylate CARHSP1 [10–13]. There is an extremely limited understanding of the function of CARHSP1 as an RNA binding protein (RBP).

Interleukin-17 (IL-17) is a critical proinflammatory cytokine that is mainly secreted by immune cells. Currently, six IL-17 family members have been identified, IL-17 A through to F [14]. IL-17 receptor (IL-17R) family includes five members, namely, IL-17RA, IL-17RB, IL-17RC, IL-17RD, and IL-17RE [15]. IL-17RA, by far the largest member of the family, is a common subunit used by multiple ligands to form functional heterodimers [16]. Recent studies have demonstrated that the IL-17 signaling pathway not only plays a vital role in autoimmune and inflammatory diseases, but also accelerates various cancer progression [17–19]. Of note, various studies have shown an increased expression of IL-17 A and IL-17RA in PCa and BPH cells [20], and IL-17 promotes PCa growth and metastasis even under castration conditions [21, 22]. On the other hand, Kiełb P. et al. reported that IL-17 A and IL-17RA expressed in both prostate tissue and lymph node metastases, highlighting the potential significance of IL-17 A and IL-17RA in PCa metastasis and premetastatic niche formation [23]. However, how the IL-17 A/IL-17RA pathway is regulated in PCa is still not clear.

In the present study, we analyzed the expression of CARHSP1 in PCa and its association with cancer progression and patient prognosis; moreover, we detected function of CARHSP1 in cancer cells in vitro and in vivo. According to bioinformatic analysis, we investigated whether CARHSP1 modulated IL-17 pathway through binding with IL-17RA mRNA as an RBP, and further confirmed the function of CARHSP1/IL-17RA axis in cell proliferation, cell invasion and immune escape. Collectively, these findings provide evidence for CARHSP1/IL-17RA axis as a novel therapeutic target for PCa.

## Materials and methods

### Cell culture and reagents

The human prostate cancer cell lines (C4-2, 22Rv1, PC-3, DU145, and LNCaP cells) and the normal prostate cell lines (RWPE-1, P69) were purchased from the American Type Culture Collection (ATCC, Manassas, VA). The cell lines mentioned above (except for RWPE-1) were cultured in RPMI 1640 medium (Sigma-Aldrich, Wisconsin, USA), containing 10% fetal bovine serum and 1% penicillin/streptomycin (Invitrogen Co., Carlsbad, CA, USA). The RWPE-1 cells were routinely cultured in keratinocyte-SFM medium. All cells were cultured at 37 °C in a humidified incubator containing 5% CO_2_ and collected at the peak of the logarithmic growth phase for experiments. Recombinant Human IL-17/IL-17 A was purchased from R&D Systems (7955-IL). Actinomycin D (Act D) was purchased from Selleck (S8964). All reagents above were reconstituted and stored following the protocol.

### Plasmid transfection and lentiviral infection

The lentiviral vector which encodes short hairpin RNA (shRNA) targeting CARHSP1 and scramble control shRNA, was constructed by GeneCopoeia (Guangzhou, China). PCa were infected by lentiviral shRNAs to generate stable cell lines with CARHSP1 knockdown. In brief, target-specific shRNA vectors and lentivirus packaging plasmids were co-transfected into 293T cells using X-tremeGENE HP DNATransfection Reagent (Roche, Switzerland) according to the manufacturer’s instructions. After 72 h transfection, viruses were harvested and further used to infect the PCa cells. Positive cells were selected with puromycin.

### Western blotting analysis

After treatment with specific experimental conditions, the total proteins were isolated with RIPA lysis buffer (P0013B, Beyotime, Shanghai, China) with a protease inhibitor, phosphatase inhibitor and 0.1 M PMSF (ST506, Beyotime). Quantification was performed with a BCA Protein Assay Kit (P0012, Beyotime). After boiling for 10 min at 100 °C, aliquots of 20 μg protein were separated by electrophoresis on 10% or 12% sodium dodecyl sulphate-polyacrylamide gels, transferred to polyvinylidene difluoride membranes, and then blocked with 5% skim milk for 1 h at room temperature. After incubating with primary antibody on a shaker at 4 °C overnight, washing three times with TBST and incubating with the corresponding secondary antibody for 2 h at room temperature, immunoblots on the membranes were visualized using an enhanced chemiluminescence detection kit (Millipore, Burlington, MA, USA). The following antibodies were used in the experiment: Antibodies against CARHSP1 (sc-137072), JAK2 (sc-390539), STAT3 (sc-8019), Phospho-STAT3 (sc-8059) were purchased from Santa Cruz Biotechnology. Antibodies against CDK4 (12790), Cyclin D3 (2936), E-cadherin (3195), N-cadherin (13116), Snail (3895), Phospho-Jak2 (Tyr1007) (4406), NF-κB p65 (8242), Phospho-NF-κB p65 (Ser536) (3033), β-actin (3700), and Vinculin (4650) were purchased from Cell Signaling Technology (Beverly, MA, USA). Antibodies against MMP9 (ab38898), Twist (ab50887), and PD-L1 (ab213524) were purchased from Abcam (Cambridge, UK). Antibodies against MMP2 (10373-2-AP) were purchased from Proteintech Group. Antibody against Human IL-17 RA/IL-17 R (133617) was purchased from R&D Systems.

### MTT assays

3-(4,5-dimethylthiazol-2-yl)-2,5-diphenyltetrazolium bromide (MTT) assay was used to evaluate the cell viability. At the end of incubation, both cell types (4000 cells per well) were incubated with MTT (5 mg/mL) (Abcam) for an additional 4 h. Then the crystal formazan was dissolved in dimethyl sulfoxide (DMSO; 150 μL/well; ECHO Chemical Co. Ltd., Shanghai), and 96-well microplate reader (Bio-Rad, Hercules, USA) was used to measure the absorbance at 490 nm.

### Colony formation assay

PCa cells were inoculated in 6-well plates containing 1000 individual cells per well in triplicate. After 2 weeks of incubation, the cells were fixed with 4% paraformaldehyde for 15 min and then stained with crystal violet for 20 min at room temperature. After the plates were air-dried, the number of colonies was determined.

### Wound healing assay

The cells were seeded into 6-well plates with the marker lines pre-draw across the bottom side and scratched with a 200 μL pipette tip to mark the distance when the cell density reached almost 100%. Then the cells were cultured in a serum-free medium, and an inverted microscope (Olympus, Tokyo, Japan) was used to take photographs every 4 h until the scratches were almost completely closed. This experiment was repeated in triplicate.

### Transwell assay

To investigate the migration and invasion abilities of PCa cells under given conditions (knockdown of CARHSP1 gene in 22Rv1 and PC-3 cells), migration and invasion assays were performed via Boyden chambers with an 8-μm pore size (Millipore, Germany). For the migration assay, chambers plated into 24-well plates were seeded with 3 × 10^4^ 22Rv1 cells or 4 × 10^4^ PC-3 cells suspended in 200 μL serum-free 1640 medium in the upper chamber without Matrigel, and 800 μL 1640 medium with 10% FBS was added to the lower chamber for 24 h. For the invasion assay, 60 μL Matrigel (Sigma, St. Louis, MO, USA) was added to the upper chamber and incubated in a cell incubator at 37 °C for 4 h, then 6 × 10^4^ 22Rv1 or 8 × 10^4^ PC-3 cells suspended in 200 μL serum-free culture 1640 medium were added to the upper chamber and 800 μL 1640 medium containing 10% FBS was added to the lower chamber for 48 h. After washing with PBS 3 times, fixing with 4% paraformaldehyde for 15 min and further staining with 0.1% crystal violet for 10 min, the visible cells were observed and counted under an inverted light microscope (magnification,×100) in three random fields for each chamber.

### RNA extraction and RT-qPCR

In brief, total RNA of PCa cells was extracted with RNAfast 200 reagents (Feijie Biotechnology, Shanghai, China) according to the manufacturer’s instructions. 2 μL of extracted RNA was used for RNA quantification by absorbance at 260 nm and was reverse transcribed to complementary DNA using a Prime Script RT-PCR kit (Takara Bio Dalian, China). Next, cDNA was amplified using specific primers using a CFX96 Real-Time PCR system (Bio-Rad, CA, USA) with SYBRGreen PCR Master Mix (Takara Bio, Dalian, China). Corresponding primer sequences are listed in Supplementary Table S1. Gene mRNA expression levels were assessed by the 2^−ΔΔCt^ method. 18 S was used for normalization.

### Animal experiments

All animal experiments were approved by the Ethical Committee of the First Affiliated Hospital of Medical College, Xi’an Jiaotong University, Xi’an, China. And care for them was in accordance with guidelines of Institutional Animal Care and Use Committee of Xi’an Jiaotong University.

For RM-1 cell xenograft model, sixteen 4-week-old male C57BL/6 mice were randomly separated into two groups with each group containing 8 mice. *Carhsp1*-knockdown or control mouse RM-1 cells (shCar and shCon) were suspended and counted in FBS-free RPMI, and 2 × 10^5^ cells were injected into mice subcutaneously. The tumor volume and mouse weights were recorded and measured every other day until the nude mice were euthanized at day 14. The harvested tumors were weighed and then stained by immunohistochemistry. The tumor volume was calculated as length×width^2^ × 1/2.

For tail vein cancer metastasis model, twelve 4-week-old male nude mice were randomly separated into two groups with each group containing 6 mice. Briefly, male nude mice were injected i.v. via the tail vein with 5 × 10^6^ 22Rv1 sublines (shCon and shCARHSP1). Then, the mouse weights were recorded and measured every three days until the nude mice were euthanized at day 46. The metastases were examined by hematoxylin-eosin (HE) staining.

### Histology and immunohistochemistry assay

Xenograft tumor and lung samples from mice were collected, fixed, embedded into paraffin, and sectioned for HE staining and immunohistochemistry according the standard protocols. Specifically, the tissue sections were deparaffinized, rehydrated, and then subject to antigen retrieval by boiling in 10 mM sodium citrate (pH6.0) for 30 min. Next, the slide was incubated with 3% H_2_O_2_ for 30 min and blocked in fetal bovine serum for 1 h at room temperature. Then the slides were incubated with specific primary antibodies at 4 °C overnight in a humidified chamber and incubated with a biotinylated secondary antibody for 1 h at room temperature the following day. Stained sections were detected using a commercial DAB chromogen Kit (GeneTech, GK800511) and finally dehydrated and mounted using Permount. The following primary antibodies were used for immunohistochemistry: anti-CARHSP1 (1:100, Abcam, ab96677), anti-Ki-67 (D3H5) (1:400, Cell Signaling Technology, 9129), anti-phospho-STAT3 (Tyr705) (D3A7) (1:200, Cell Signaling Technology, 9145), anti-PD-L1 (D5V3B) (1:100, Cell Signaling Technology, 64988), and anti-CD8 alpha (1:100, Abcam, ab316778). Three randomly selected fields per mouse were captured and the results were evaluated based on the intensity of staining (0, 1, 2, and 3) and the proportion of positive cells (0 (no positive cells), 1 (< 10% of positive cells), 2 (10–50% positive cells), 3 (51–80% positive cells), and 4 (> 80% positive cells)). Final immunoreactive score (IRS) was calculated as staining intensity×positive cells proportion.

### Flow cytometry

After treatment with the given conditions, cells were collected and washed twice with precooled PBS. Subsequently, cells were treated with different reagents. To detect IL-17RA or PD-L1 on PCa cell surface, the following primary antibodies were incubated with the cells for 30 min at 4 °C in PBS: Human IL-17 RA/IL-17 R Antibodies (R&D Systems, 133617); CD274 (PD-L1, B7-H1) Monoclonal Antibody (MIH1 clone, eBioscience, 14-5983-82). After incubation, cells were washed twice with PBS and incubated with APC-conjugated anti-mouse IgG1 Antibody (Biolegend, 406609) in PBS for an additional 30 min at 4 °C in the dark. To detect PD-1 on Jurkat cell surface, cells were incubated with FITC-conjugated anti-human CD279 (PD-1) Antibody (Biolegend, 329903) in PBS for 30 min at 4 °C in the dark. To measure cell death, the cells (including floating dead cells) were stained with 5 μg/mL propidium iodide (PI) (Roche) for 15 min at room temperature in dark. Finally, the cells were washed with PBS three times after staining and detected by a FACSCalibur™ flow cytometer (BD Biosciences, Franklin Lakes, NJ, USA) to assess the staining signals, and flow cytometry data were analyzed utilizing FlowJo software (v10.8.1). The experiment was repeated three times.

To analyze the apoptotic ratio of cells, the cells were resuspended in 100 μL of binding buffer, and mixed with 4 μL of Annexin V-FITC and 4 μL PI for 15 min in dark. After that, 400 μL of binding buffer was added for dilution and the staining signals of cells was assessed by the same method as above.

### mRNA stability assay

PCa cells transfected with shCAR or shCon were treated with 5 μg/mL Actinomycin D (S8964, Selleck) to inhibit mRNA transcription. Total RNA was extracted for cDNA synthesis and detected by RT-qPCR as indicated. Gene mRNA expression levels were assessed by the 2^−ΔΔCt^ method. GAPDH was used for normalization. Relative mRNA levels were normalized to the starting point of treatment.

### RNA pull-down and silver staining

PCa cells in a 100-mm dish were collected in 1mL of cell lysis buffer at 4 °C. The cell lysates were centrifuged at 12,000 g for 15 min. The supernatant was incubated with biotinylated IL-17RA mRNA and non-specific binding RNA (NS RNA, used as negative controls) at 37 °C overnight. The next day, 25 μL streptavidin-conjugated magnetic beads (ThermoFisher Scientific, USA) was added and incubated for at least 1 h at 4 °C. Then streptavidin beads were collected and washed with lysis buffer five times. The bound proteins were collected by adding 30 μL 1.5×SDS buffer. CARHSP1 protein was detected by western blotting and silver staining. All sequences of RNA are summarized in the Supplementary Table S2.

### T cell-mediated tumor cell killing assay

Jurkat cells were pre-activated for 24 h with STEMCELL Technologies’ stimulating reagents ImmunoCult™ Human CD3/CD28/CD2 T Cell Activator (25 μL/mL, 10970) in RPMI1640 supplemented with 10% FBS. The expression levels of PD-1 was assessed by flow cytometry to evaluate whether the Jurkat cells were effectively activated. 22Rv1 and PC-3 cells with CARHSP1 stably knockdown were inoculated into 6-well plates at a density of 2 × 10^5^. Activated Jurkat cells were then mixed with tumor cells at a density of 1 × 10^6^ (T cell to tumor cell ratio of 5:1). After 24 h co-culture, both the adherent tumor cells and suspended Jurkat cells were collected and analyzed by flow cytometry. Jurkat cells were also collected to evaluate the expression of IFN-γ, IL-2, and TNF-α by RT-qPCR. The culture medium was collected to ELISA assay for the secretion of IFN-γ.

### ELISA

Cytokine assays were performed using Human IFN-γ (#EK180-48) ELISA kits (Multi Sciences, Hangzhou, China) according to the manufacturer’s instructions.

### mRNA expression analysis and survival analysis

We downloaded data from the TCGA and GEO databases, including GSE21034, GSE6919, GSE3325, GSE5132, GSE21035 and GSE35988 and analyzed the mRNA expression values of CARHSP1 between PCa tissues and normal tissues. Furthermore, we explored the mRNA expression levels of CARHSP1 in different clinical stages and also different metastatic conditions. The relationship between expression of CARHSP1 and progression-free survival (PFS) rate or disease-free interval (DFI) rate were analyzed with GSCA database (http://bioinfo.life.hust.edu.cn/GSCA/#/). The mRNA expression levels of CARHSP1 between disease-free PCa tissues and recurred PCa tissues were analyzed with CANCERTOOL database (http://web.bioinformatics.cicbiogune.es/CANCERTOOL/).

### Statistical analysis

Briefly, the differences between two groups were analyzed by Student’s t-test implemented with GraphPad Prism 7.0 software (GraphPad, CA, USA). The data are presented as the mean ± standard deviation (SD) of three independent experiments. *P* < 0.05 was regarded as the threshold value for statistical significance.

## Results

### Expression of CARHSP1 increased in prostate cancer progression, and its upregulation is associated with shorter survival of patients

**Fig. 1:**
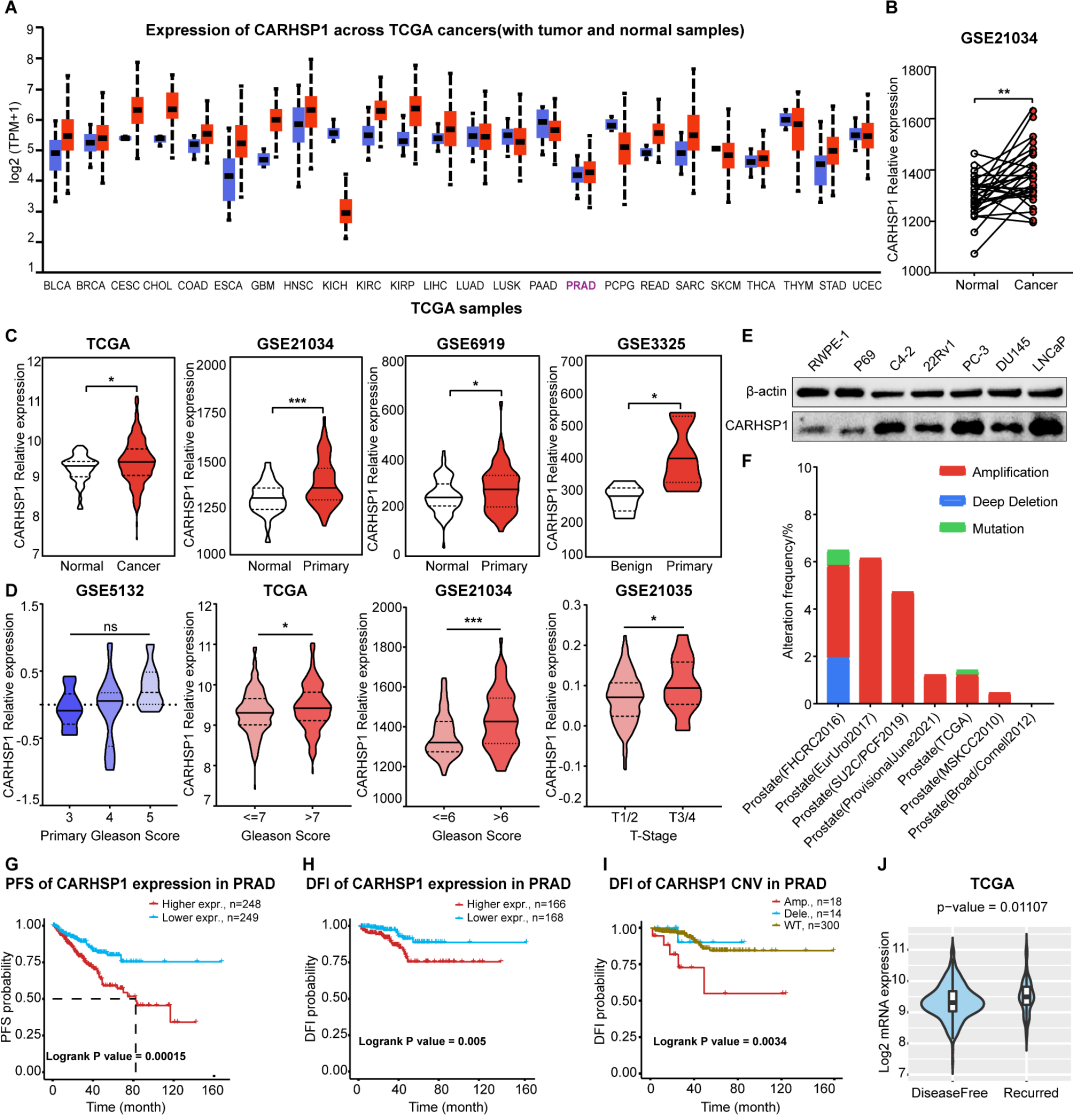
CARHSP1 is upregulated and associated with a worse prognosis in PCa. (**A**) CARHSP1 expression in various cancers and corresponding normal tissues from the UALCAN cancer database. Red column, cancer tissues; blue column, normal tissues. (**B**) CARHSP1 transcript levels in paired normal prostate tissues (*n* = 29) and prostate cancer tissues (*n* = 29) from GEO (GSE21034) databases. (**C**) Violin plots demonstrating CARHSP1 mRNA overexpression in PCa primary/malignant tissues compared to normal/benign tissues in four independent datasets. (**D**) CARHSP1 mRNA expression in prostate cancer tissues with different Gleason score and diseases stage (T-stage). Data were extracted from TCGA and GEO PCa datasets. (**E**) Western blotting analysis of CARHSP1 protein expression levels in human prostate cancer cell lines (C4-2, 22Rv1, PC-3, DU145, and LNCaP cells) and the normal prostate cell lines (RWPE-1, P69). β-actin was used as the internal loading control. (**F**) Analysis of CARHSP1 mutation and copy-number alterations frequency across 7 PCa genomic datasets in cBioportal website. **(G**,** H**) Association between progression-free survival (PFS)/disease-free interval (DFI) of prostate cancer patients and CARHSP1 mRNA expression from the GSCA PCa database. (**I**) Association between DFI of prostate cancer patients and CARHSP1 copy number variation from the GSCA PCa database. (**J**) CARHSP1 expression in primary and recurrent PCa tissues from TCGA database. *p < 0.05; **p < 0.01; ***p < 0.001; ns, not significant

Initially, bioinformatics analysis on the ULCAN website showed that CARHSP1 was highly expressed in several cancer types (Fig. 1A). Then we analyzed CARHSP1 expression in PCa by using RNA-sequencing data from TCGA database and found that CARHSP1 expression was significantly higher in malignant compared to normal prostate tissues. This result was also validated in three independent GEO expression datasets of PCa tissues versus non-malignant tissues, one of which offered data of paired PCa and normal prostate tissues (Fig. 1B and C). Further analysis of the TCGA data and GEO data revealed increased CARHSP1 expression correlated with increased Gleason score or with advanced disease stage. Although not statistically significant, CARHSP1 expression in primary Gleason score showed the same trend (Fig. 1D). Then, we further examined CARHSP1 expression in a panel of prostate cancer cell lines (C4-2, 22Rv1, PC-3, DU145, and LNCaP cells) and the normal prostate epithelial cell lines (RWPE-1, P69) by the western blotting analysis, and found that CARHSP1 expression was markedly increased in all five tested prostate cancer cell lines compared with two non-malignant prostate cell lines (Fig. 1E; Fig. S1A). Moreover, consistent with our observation of increased CARHSP1 expression in PCa, CARHSP1 amplification was evident in several clinical datasets accessed via cBioPortal (Fig. 1F). On the other hand, both progression-free survival (PFS) and disease-free interval (DFI) of prostate cancer patients in the CARHSP1 high-expression group were significantly shorter than in the CARHSP1 low-expression group from the GSCA PCa database (Fig. 1G and H). In particular, the association between CARHSP1 mRNA expression and DFI became more obvious after further classified according to the copy number variation (Fig. 1I). Furthermore, we also found that CARHSP1 mRNA expression in recurrent PCa tissues was dramatically increased compared with primary PCa tissues in the TCGA dataset (Fig. 1J). Taken together, these findings demonstrate that expression of CARHSP1 is closely associated to PCa progression and patient outcomes, suggesting that CARHSP1 might be a potential therapeutic target in PCa.

### CARHSP1 inhibition decreased PCa cell proliferation

**Fig. 2:**
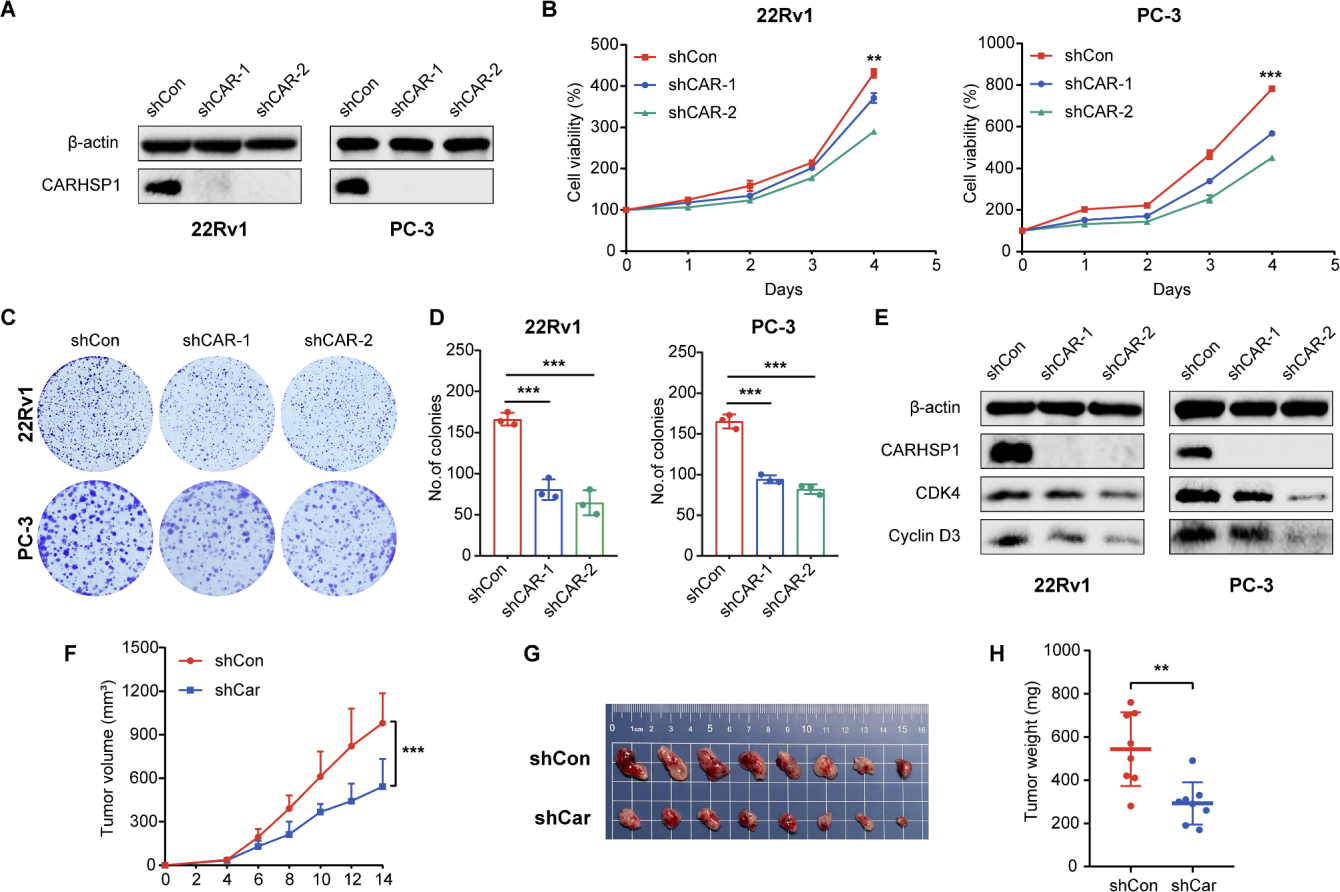
CARHSP1 inhibition decreases PCa cell proliferation in vitro and in vivo. (**A**) Western blotting analysis of CARHSP1 protein levels in 22Rv1 and PC-3 cells transfected with CARHSP1 shRNAs (shCAR) or negative control (shCon). β-actin was used as the internal loading control. (**B**) Cell viability in 22Rv1 and PC-3 cells transfected with shCAR or shCon detected by an MTT assay. **(C**,** D**) Representative images of the colony formation assay in 22Rv1 and PC-3 cells transfected with shCAR or shCon and quantification analyses of the colony number. (**E**) Western blotting analysis of cell cycle-related proteins CDK4 and Cyclin D3 levels in 22Rv1 and PC-3 cells transfected with shCAR or shCon. β-actin was used as a loading control. (**F**) The growth curves of RM-1 cell xenografts in the shCon group and shCar group (*n* = 8 per group). Tumor volumes were measured every other day. (**G**) Representative image of the tumors obtained from C57BL/6 mice in the shCon group and shCar group. (**H**) At 14 days after cells injection, the wet weights of tumors excised from each group of mice were measured. Data are presented as means (± SD), *n* = 3 independent repeats. Unpaired, two-tailed t test. **p < 0.01; ***p < 0.001

To gain insight into the biological functions of CARHSP1 in PCa, we employed gene set enrichment analysis (GSEA) based on the TCGA PCa cohort and hallmark signatures, and found that the gene set of “Hallmark_G2m Checkpoint” was significantly positively associated with CARHSP1 mRNA levels (Fig. S1B). Combined with the results that high CARHSP1 levels correlate with higher T stages (Fig. 1), these results uncovered that prostate cancer with high CARHSP1 levels may exhibit fast cell proliferation. To confirm the potential oncogenic function of CARHSP1 in PCa progression, we designed two short hairpin RNA (shCAR-1 and shCAR-2) to knock down the endogenous expression of CARHSP1 in PCa cells and the efficiency of knockdown was validated by western blotting analysis (Fig. 2A; Fig. S1C). The successfully established stable sublines were used for subsequent experiments. Functionally, MTT assays revealed that CARHSP1 knockdown significantly impeded the proliferation of PCa cells (Fig. 2B; Fig. S1D). Additionally, colony formation assays provided further confirmation of the enhancing effect of CARHSP1 on the growth of prostate cancer cells (Fig. 2C and D; Fig. S1 E, S1F). Meanwhile, the expression of cell cycle-related genes such as CDK4, and Cyclin D3 was decreased after CARHSP1 knockdown (Fig. 2E). These findings indicate that CARHSP1 loss could suppress PCa cell proliferation capacity in vitro. To verify the tumorigenicity role of CARHSP1 in vivo, a xenograft model was established. *Carhsp1*-knockdown or control mouse RM-1 cells were established (Fig. S1G, S1H) and injected into C57BL/6 mice (immune competent). Tumor volumes and body weights were measured every other day until the mice were euthanized at day 14. There was no significant change in body weight between the two groups (Fig. S1I). It was clearly displayed that *Carhsp1* knockdown can inhibit xenograft growth in vivo (Fig. 2F, G and H). All results suggested that CARHSP1 knockdown attenuated the tumor proliferation of PCa in vitro and in vivo.

### Knockdown of CARHSP1 attenuated the migration and invasion ability of prostate cancer cells in vitro and in vivo

**Fig. 3:**
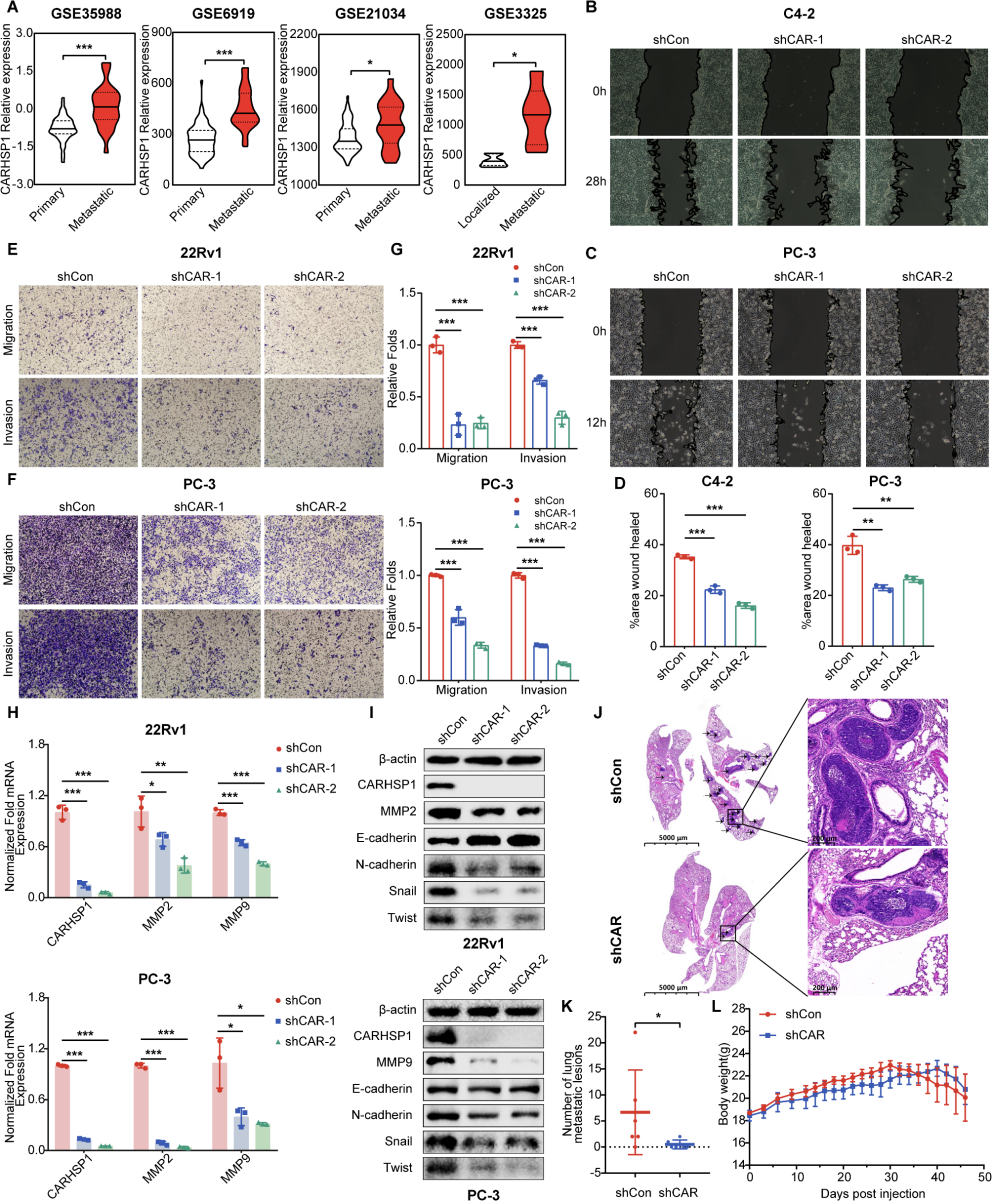
Knockdown of CARHSP1 attenuates the migration and invasion ability of prostate cancer cells. (**A**) Violin plots showing CARHSP1 mRNA overexpression in metastatic PCa tissues compared to primary/localized PCa tissues in four independent GEO datasets. (**B-D**) Representative images of wound healing assays in C4-2 and PC-3 cells transfected with shCAR or shCon and quantitative data (*n* = 3, mean ± SD). (**E-G**) Representative images of transwell assays and quantification analysis of migration and invasion ability in 22Rv1 and PC-3 cells transfected with shCAR or shCon (*n* = 3, mean ± SD). (**H**) Effect of CARHSP1 knockdown on the expression of invasion-associated genes *MMP2* and *MMP9* in 22Rv1 and PC-3 cells, as detected by RT-qPCR (*n* = 3, mean ± SD). 18 S was used as an internal loading control. (**I**) Western blotting analysis of metastasis and EMT biomarkers levels after CARHSP1 knockdown in 22Rv1 and PC-3 cells. β-actin was used as a loading control. (**J**) The histological changes detected by HE staining of the lungs from the 22Rv1/shCon and 22Rv1/shCAR groups. Arrows: foci of lung metastasis. (**K**) Quantification analyses of the number of nude mice lung metastatic lesions in 22Rv1/shCon and 22Rv1/shCAR groups. (**L**) Quantification analyses of the changes of nude mice body weight in 22Rv1/shCon and 22Rv1/shCAR groups. *p < 0.05; **p < 0.01; ***p < 0.001

Higher CARHSP1 mRNA expression in metastatic tumor tissues than in localized tumor tissues of PCa was evidenced by the analysis from four independent GEO expression datasets (Fig. 3A). Additionally, GSEA showed that “Hallmark_Epithelial Mesenchymal Transition” (EMT) pathway was enriched in the CARHSP1 high expression group, which is the leading cause of distant metastasis (Fig. S1B). Correlation analysis in prostate cancer samples from TCGA cohort using Tumor Immune Estimation Resource (TIMER) and Gene Expression Profiling Interactive Analysis (GEPIA) database demonstrated a positive correlation between mRNA levels of CARHSP1 and MMP2, MMP9, CDH2 (N-cadherin), SNAI1 (Snail), and VIM (vimentin) (Fig. S2A, S2B). Functionally, knockdown of CARHSP1 weakened the migration and invasion capacity of prostate cancer cells as detected by wound healing assays (Fig. 3B, C and D; Fig. S2C, S2D, S2E) and transwell assays (Fig. 3E, F and G). Moreover, it was observed that CARHSP1 increased the expression of MMP2 and MMP9 at the mRNA level and the protein level, which play vital roles in tissue remodeling, cancer progression, and metastasis (Fig. 3H and I; Fig. S2F). Further examination of the influence of CARHSP1 on EMT markers showed that CARHSP1 knockdown blocked EMT by simultaneously upregulated an epithelial marker (E-cadherin) and downregulated mesenchymal markers (N-cadherin, Snail, and Twist) in C4-2, DU145, 22RV1, and PC-3 sublines (Fig. 3I; Fig. S2F).

To verify whether CARHSP1 could promoting the prostate cancer metastasis in vivo, the 22RV1 sublines (22Rv1/shCon and 22Rv1/shCAR groups) were used to establish the tail-vein injection metastasis mouse models. Hematoxylin-eosin (HE) staining showed that nude mice receiving the 22Rv1/shCon control group formed a higher number of lung metastatic foci compared with 22Rv1/shCAR subline (*p* < 0.05; Fig. 3J and K; Fig. S2G). Lung weight in 22Rv1/shCAR group was also slightly lower than in 22Rv1/shCon group (Fig. S2H). However, there was no significant change in body weight between the two groups (Fig. 3L). Taken together, our data indicates that CARHSP1 loss could inhibit PCa metastasis in vitro and in vivo.

### CARHSP1 directly enhanced IL-17RA mRNA stability by binding to the 3’-UTR of IL-17RA as an RBP

**Fig. 4:**
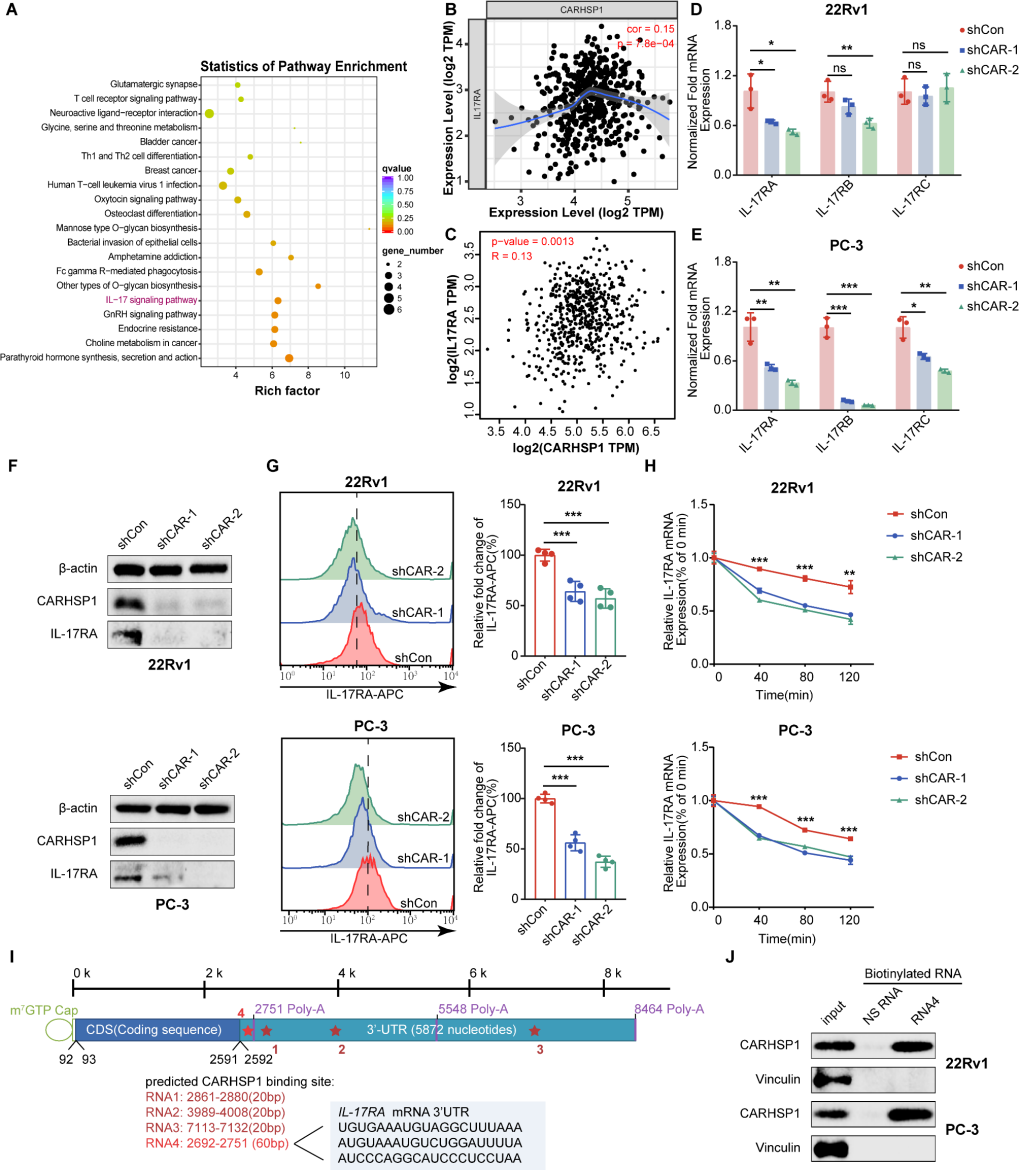
CARHSP1 directly regulates IL-17RA mRNA stability by binding to the 3’-UTR of IL-17RA. (**A**) KEGG pathway enrichment analysis of differentially expressed genes in 22Rv1 cells transfected with shCAR or shCon from the RNA-seq data. **(B**,** C**) Correlation analysis between mRNA expression levels of CARHSP1 and IL-17RA in PCa based on the TCGA cohort using TIMER (B) and GEPIA (C) database. **(D**,** E**) Effect of CARHSP1 knockdown on the expression of IL-17 receptor family members *IL-17RA*, *IL-17RB* and *IL-17RC* in 22Rv1 and PC-3 cells, as detected by RT-qPCR (*n* = 3, mean ± SD). 18 S was used as an internal loading control. (**F**) Western blotting analysis of the change of IL-17RA protein levels after CARHSP1 knockdown in 22Rv1 and PC-3 cells. β-actin was used as a loading control. (**G**) Cell surface analysis of IL-17RA protein using flow cytometry in 22Rv1 and PC-3 cells with stable depletion of CARHSP1 (*n* = 4, mean ± SD). (**H**) Measurement of *IL-17RA* mRNA stability by RT-qPCR after 22Rv1 and PC-3 cells transfected with shCAR or shCon were exposed to Actinomycin D (Act D) (5 μg/mL). The data are expressed as the percentage of mRNA molecules before the Actinomycin D treatment (*n* = 3, mean ± SD). (**I**) Diagram of the predicted CARHSP1 binding sites on the *IL-17RA* mRNA 3’ untranslated region (3’-UTR). (**J**) Analysis of the interaction of CARHSP1 with the *IL-17RA* 3’-UTR by RNA pull-down assays followed by western blot analysis in 22Rv1 and PC-3 cells. Vinculin was used as a loading control. NS RNA were random, non-specific binding RNAs that were used as negative controls. *p < 0.05; **p < 0.01; ***p < 0.001; ns, not significant

To further understand the molecular basis for CARHSP1 on PCa progression, we performed RNA sequencing (RNA-seq) to detect differentially expressed genes following knockdown of CARHSP1 in 22Rv1 cells. According to the test reports, KEGG pathway enrichment analysis revealed that the IL-17 signaling pathway might be involved in CARHSP1-induced PCa growth and metastasis (Fig. 4A). As IL-17RA plays an important role in the IL-17 signaling pathway, we performed a correlation analysis of CARHSP1 and IL-17RA mRNA expression levels derived from the TCGA PCa cohort using TIMER database and GEPIA database. The consistent results indicated that the mRNA expression levels of CARHSP1 were positively correlated with IL-17RA (Fig. 4B and C). Then we measured the mRNA levels of IL-17RA, IL-17RB, and IL-17RC after knocking down CARHSP1 in PCa cells by RT-qPCR. Only the correlation between CARHSP1 and IL-17RA was always significant across all PCa cells and the two knockdown sequences, indicating that CARHSP1 specifically regulated the level of IL-17RA (Fig. 4D and E; Fig. S3A). Moreover, we further verified that knockdown of CARHSP1 resulted in the reduction of IL-17RA expression at the protein level by western blotting analysis and flow cytometry analysis in 22Rv1 and PC-3 cells (Fig. 4F and G). As mentioned before, it has been reported that CARHSP1 functions as a TNF-α mRNA stability enhancer, which is necessary for effective TNF-α production [7–9]. Therefore, we then detected the role of CARHSP1 in TNF-α regulation in prostate cancer cells using RT-qPCR assay. However, the result indicated that the effect of CARHSP1 knockdown on *TNF-α* mRNA was not consistent among different prostate cancer cell lines, which suggests that there are other factors in prostate cancer cells interfering with the regulation of CARHSP1 on TNF-α (Fig. S3B).

Considering CARHSP1 contains a CSD domain with two RNA binding motifs in its protein structure, it is worth exploring whether CARHSP1 could directly regulate IL-17RA mRNA stability by binding to the 3’-UTR of IL-17RA as an RNA-binding protein. Knockdown of CARHSP1 significantly reduced the half-life of IL-17RA mRNA in PCa cell lines after treatment with actinomycin D (Act D), as detected by RT-qPCR (Fig. 4H; Fig. S3C). Moreover, to predict potential RNA-protein interactions, we used the RBPsuite to predict the possible binding sites of CARHSP1 on IL-17RA mRNA. Unfortunately, CARHSP1 protein is not included in database, but we found IL-17RA mRNA were predicted to bind with YBX3 protein, with several possible binding sites suggested (Fig. S3D). CARHSP1 and YBX3 both belong to the family of cold shock domain proteins, which is characterized by the presence of an evolutionarily conserved cold shock domain and exerts pleiotropic functions in cells via their ability to bind single-stranded RNA and/or DNA. We also utilized the online prediction tools catRAPID and RPISeq websites, which predicted a relatively high score of IL-17RA mRNA-CARHSP1 interaction (Fig. S3E; Fig. S3F). To further verify the predicted results, RNA pull-down experiments were carried out in PCa cells. It is well-characterized that most RNA-binding proteins stabilize their mRNA targets through direct binding to their specific cis-acting elements in the 3’-UTRs, such as AU-rich element (ARE). We identified 3 potential AREs (named RNA1, RNA2, and RNA3) in the 3’-UTR of IL-17RA via online bioinformatic tools (AREsite2). As the positions of all three sequences follow the first polyA site, we designed RNA4 preceding the first polyA site (Fig. 4I). To analyze the binding, we generated biotin-labeled short RNA sequences and then pulled down the RNA and protein complex in cell lysates with streptavidin beads. As detected by western blotting analysis, CARHSP1 protein was only detected in the cell lysate immunoprecipitated with biotinylated RNA4 rather than RNA1, RNA2, or RNA3 (Fig. 4J; Fig. S3G, S3H). Silver staining were also performed and the results revealed several potential proteins binding to the 3’-UTR of IL-17RA, including protein whose molecular weight is 20KD (Fig. S3I). Although the protein is most likely CARHSP1, the final validation of the involved protein requires MS analysis. Collectively, these results demonstrate that CARHSP1 can enhance IL-17RA mRNA stability by binding to the 3’-UTR of IL-17RA directly.

### CARHSP1 promoted the proliferation, migration, and invasion of PCa cells via IL-17RA/STAT3 signaling

**Fig. 5:**
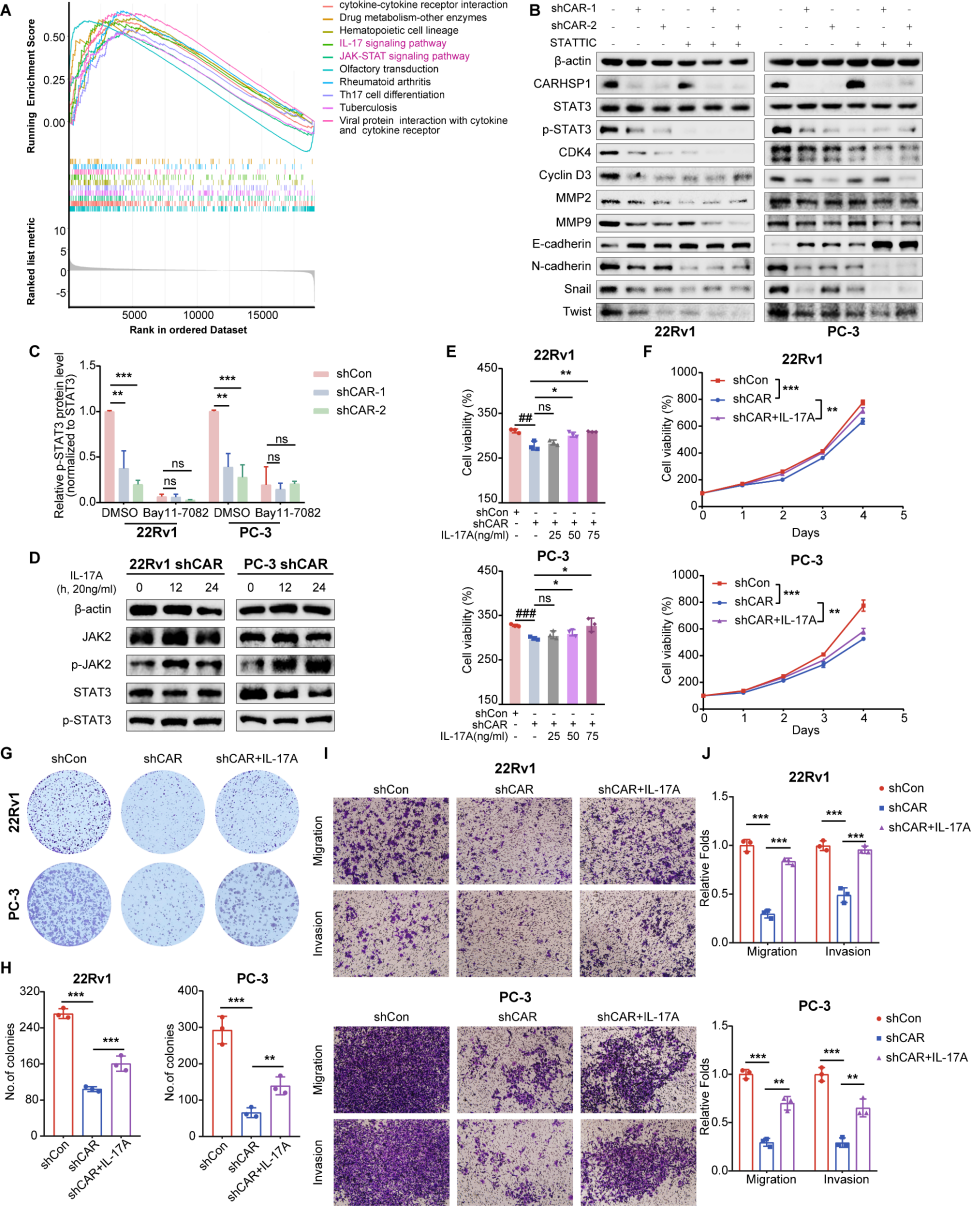
CARHSP1 promotes the proliferation, migration, and invasion of PCa cells via IL-17RA/STAT3 signaling. (**A**) GSEA plot revealing a positive correlation between CARHSP1 mRNA levels and the “IL-17 signaling pathway” and “JAK-STAT signaling pathway” in TCGA PCa dataset. **(B**,** C**) Expression of CARHSP1, STAT3, p-STAT3, and pathway-related proteins in 22Rv1 and PC-3 cells transfected with shCAR or shCon after adding inhibitor STATTIC (1 μM) for 24 h was detected by western blotting analysis. β-actin was used as a loading control. (**D**) Western blotting analysis of JAK2, p-JAK2, STAT3, and p-STAT3 in 22Rv1 and PC-3 shCAR cells after treatment with recombinant human IL-17 A (20 ng/mL). β-actin was used as a loading control. (**E**) Cell viability in 22Rv1 and PC-3 shCAR or shCon cells after treatment with recombinant human IL-17 A at different concentrations for 48 h detected by MTT assay (*n* = 3, mean ± SD). (**F**) Cell viability in 22Rv1 and PC-3 shCAR or shCon cells after treatment with recombinant human IL-17 A (50 ng/mL) detected by MTT assay (*n* = 3, mean ± SD). **(G**,** H**) Representative images of the colony formation assay in 22Rv1 and PC-3 shCAR or shCon cells after treatment with recombinant human IL-17 A (50 ng/mL) and quantification analyses of the colony number (*n* = 3, mean ± SD). **(I**,** J**) Representative images of transwell assays and quantification analysis of migration and invasion ability in 22Rv1 and PC-3 shCAR or shCon cells treated as described above (*n* = 3, mean ± SD). *p < 0.05; **p < 0.01; ***p < 0.001; ns, not significant versus the CARHSP1 shRNA group (shCAR). ## *p* < 0.01 versus the scrambled shRNA group (shCon)

**Fig. 6:**
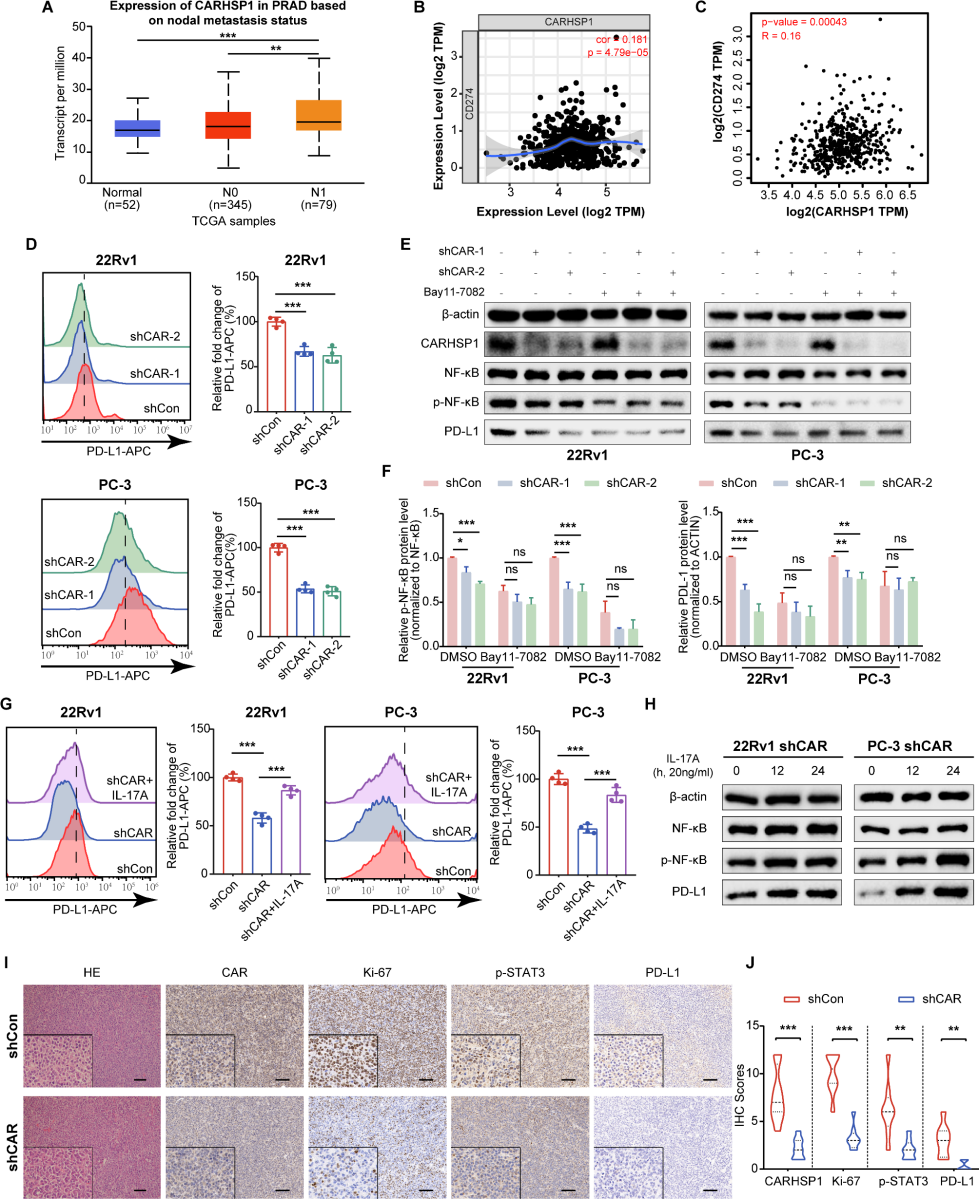
CARHSP1 upregulates PD-L1 expression by activating IL-17RA/ NF-κB signaling. (**A**) CARHSP1 mRNA overexpression in lymph node metastatic tumors (*n* = 79) compared to in primary prostate tumors (*n* = 345) or normal prostate tissues (*n* = 52) from the UALCAN cancer database. **(B**,** C**) Correlation analysis between mRNA expression levels of CARHSP1 and PD-L1 in PCa based on the TCGA cohort using TIMER (B) and GEPIA (C) database. (**D**) Analysis of cell surface PD-L1 protein using flow cytometry in 22Rv1 and PC-3 cells with stable depletion of CARHSP1 (*n* = 4, mean ± SD). **(E**,** F**) Western blotting analysis of the expression levels of CARHSP1, NF-κB, p-NF-κB, and PD-L1 in 22Rv1 and PC-3 cells transfected with shCAR or shCon after adding inhibitor Bay11-7082 (5 μM) for 24 h. β-actin was used as a loading control. (**G**) Analysis of cell surface PD-L1 protein using flow cytometry in 22Rv1 and PC-3 shCAR or shCon cells after treatment with recombinant human IL-17 A (20 ng/mL) (*n* = 4, mean ± SD). (**H**) Western blotting analysis of NF-κB, p-NF-κB, and PD-L1 in 22Rv1 and PC-3 shCAR cells after treatment with recombinant human IL-17 A (20 ng/mL). β-actin was used as a loading control. **(I**,** J**) Representative images of H&E staining and IHC analysis (CARHSP1, Ki-67, p-STAT3, and PD-L1) in the RM-1 cell xenograft tumors and quantitative data. The scale bar represents 100 μm. **p < 0.01; ***p < 0.001; ns, not significant

Since numerous studies showed that IL-17/IL-17R can trigger the release of proinflammatory cytokine IL-6 and the consequent activation of the STAT3 pathway, it was natural to wonder whether CARHSP1 would affect the STAT3 pathway in PCa cells. GSEA and KEGG pathway analysis in TCGA PCa dataset suggested that “JAK-STAT signaling pathway” was upregulated when CARHSP1 was highly expressed (Fig. 5A; Fig. S1B). Of note, the IL-17 signaling pathway was also enriched in this KEGG pathway analysis, which is consistent with the result of the KEGG pathway enrichment analysis based on RNA-seq. As detected by RT-qPCR assay, the correlation between CARHSP1 and IL-6 was always significant across all the PCa cells and the two knockdown sequences (Fig. S4A). Western blotting analysis revealed that CARHSP1 knockdown resulted in the reduction of phosphorylated JAK2 protein levels and phosphorylated STAT3 protein levels (Fig. 5B and C; Fig. S4B), which was also observed in *vivo* by immunohistochemistry assay (Fig. 6I and J). However, treatment with STATTIC (a STAT3 inhibitor) could significantly attenuate the downregulation of p-STAT3 expression and pathway-related downstream proteins expression caused by CARHSP1 knockdown and have no effect on the expression of CARHSP1, which verified that JAK-STAT3 signaling pathway was activated by CARHSP1 in PCa cells (Fig. 5B and C). To explore whether CARHSP1 maintains the activation of STAT3 and promotes PCa growth via IL-17RA, 22Rv1 and PC-3 cells were treated with recombinant human IL-17 A after CARHSP1 knockdown and western blotting analysis showed that the JAK-STAT3 signaling pathway was reactivated after IL-17 A treatment (Fig. 5D). Subsequently, functional experiments were performed to further verify the mechanism. MTT assays revealed that IL-17 A stimulation partly reversed the proliferation inhibition induced by CARHSP1 knockdown in both concentration dependent manner and time dependent manner (Fig. 5E and F). Similar results were also observed in the colony formation assay (Fig. 5G and H). With respect to the migration and invasion phenotype, transwell assays were conducted in 22Rv1 and PC-3 shCAR cells after treatment with IL-17 A, and the results showed IL-17 A stimulation partly reversed the migration and invasiveness inhibition induced by CARHSP1 knockdown (Fig. 5I and J). These results collectively provide evidence that CARHSP1 exerts its tumor promoting role and maintains the activation of STAT3 via adjusting the expression of IL-17RA in PCa.

### CARHSP1 promoted immune escape of PCa cells via the IL-17RA/NF-κB/PD-L1 signaling

In many solid malignancies, lymph node (LN) metastases resist T cell-mediated cytotoxicity, induce antigen-specific regulatory T cells, and generate tumor-specific immune tolerance, which represents a harbinger of distant metastatic disease. As shown in Fig. 3, expression of CARHSP1 was higher in PCa metastatic tissues. Furthermore, CARHSP1 mRNA expression significantly increased in node metastasis PCa, as evidenced by the analysis from the ULCAN website (Fig. 6A). Programmed death ligand 1 (PD-L1) (Cd274) on tumor surface enables the evasion of immune cells and suppression of T cells. Previous studies have found that NF-κB might be upstream in the regulation of PD-L1 expression in many types of cancer, and GSEA results identified a positive correlation between CARHSP1 levels and “Hallmark_TNF-α_signaling Via NF-κB” in PCa (Fig. S1B). We also analyzed the correlation between mRNA expression levels of CARHSP1 and chemokines and cytokines induced by NF-κB pathway in PCa based on the TCGA cohort using TIMER database. The results showed that NF-κB pathway target IL-6, IL-1β, C-X-C motif ligand 1 (CXCL1), and C-C motif ligand 20 (CCL20) were positively associated with the CARHSP1 expression, which further confirmed the activation of NF-κB pathway (Fig. S4C). We found that CARHSP1 increased the expression of IL-6 (Fig. S4A). However, the relationship between the CARHSP1 and PD-L1 in prostate cancer is still unknown. Firstly, correlation analysis in prostate cancer samples from TCGA cohort using TIMER and GEPIA database showed a positive correlation between mRNA levels of CARHSP1 and PD-L1 (Fig. 6B and C). To validate the results of TCGA analysis, we performed western blotting analysis and flow cytometry analysis and found that the level of PD-L1 was reduced significantly after CARHSP1 depletion in 22Rv1, PC-3, and DU145 cells (Fig. 6D, E and F; Fig. S4D, S4E). However, treatment with Bay11-7085 (a NF-κB inhibitor) could significantly attenuate the downregulation of p-p65 and PD-L1 expression caused by CARHSP1 knockdown, which suggest that the decreased level of p-p65 was at least partially responsible for the impact of CARHSP1 on PD-L1 expression in PCa cells (Fig. 6E and F). To assess the role of IL-17RA signaling pathway in regulating NF-κB/PD-L1 axis in PCa cells, 22Rv1 and PC-3 cells were treated with IL-17 A after CARHSP1 knockdown. Both western blotting analysis and flow cytometry assay showed that the NF-κB/PD-L1 axis was reactivated after IL-17 A treatment in 22Rv1 and PC-3 shCAR cells (Fig. 6G and H). In particular, immunohistochemistry assay indicated that PD-L1 were downregulated after CARHSP1 knockdown in RM-1 cell xenograft tumors (Fig. 6I and J).

**Fig. 7:**
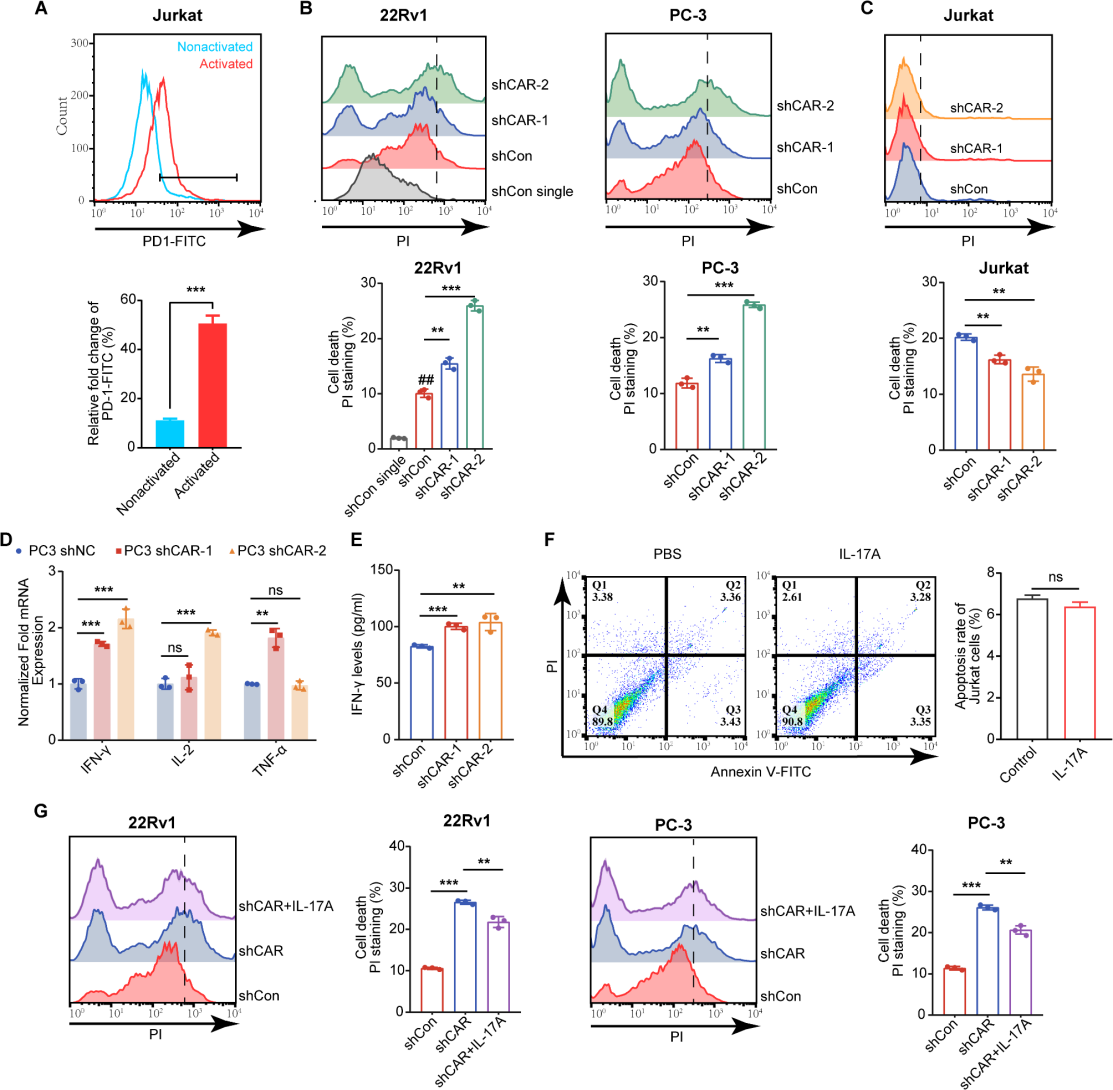
CARHSP1 knockdown stimulates anti-tumor T cell immunity. (**A**) Analysis of cell surface PD-1 protein using flow cytometry in Jurkat cells after the treatment with CD3/CD28/CD2 T Cell Activator (25 μL/mL) for 24 h (*n* = 3, mean ± SD). **(B**,** C**) T cell-mediated tumor cell killing assay in 22Rv1 and PC-3 cells with stable depletion of CARHSP1. Activated Jurkat cells were co-cultured with control or CARHSP1-depleted PCa cells at the T cell to tumor cell ratio of 10:1. After 24 h interaction, cell death of both PCa cells (B) and Jurkat cells (C) in the PCa cell-Jurkat cell co-culture systems were analyzed by PI staining followed by flow cytometry. The quantitative ratio of dead cells is showed by the bar graph (*n* = 3, mean ± SD). (**D**) The secretion of IFN-γ, IL-2, and TNF-α by Jurkat cells after co-cultured with PC-3 cells transfected with shCAR or shCon for 24 h, as detected by RT-qPCR (*n* = 3, mean ± SD). 18 S was used as an internal loading control. (**E**) The levels of IFN-γ produced by Jurkat cells after co-cultured with PC-3 cells for 24 h, as detected by ELISA (*n* = 3, mean ± SD). (**F**) Apoptosis of Jurkat cells after treatment with IL-17 A (20 ng/mL), as detected by flow cytometry assay (*n* = 3, mean ± SD). (**G**) T cell-mediated tumor cell killing assay in 22Rv1 and PC-3 cells with stable depletion of CARHSP1 after treatment with IL-17 A (20 ng/mL). Activated Jurkat cells were co-cultured with control or CARHSP1-depleted PCa cells as described above. Simultaneously treatment with IL-17 A (20 ng/mL) for 24 h, cell death of both PCa cells in the PCa cell-Jurkat cell co-culture systems was analyzed by PI staining followed by flow cytometry. (*n* = 3, mean ± SD). **p < 0.01; ***p < 0.001; ns, not significant

To investigate the potential role of CARHSP1 in tumor immunity of PCa, an in vitro co-culture model of Jurkat cells and PCa cells was established. The Jurkat cells were preactivated with CD3/CD28/CD2 T cell activator for 24 h, and the enhanced expression of PD-1 as evidenced by flow cytometry analysis confirmed that Jurkat cells were effectively activated (Fig. 7A). Subsequently, PCa cells were co-cultured with Jurkat cells for 24 h, and the death of PCa cells and Jurkat cells in co-culture system were analyzed by flow cytometry with PI staining. Knock down of CARHSP1 in 22Rv1 and PC-3 cells enhanced the T cell-mediated cancer cell killing with increased death PCa cells (Fig. 7B). In the meantime, due to CARHSP1-mediated upregulation of PD-L1 affecting T cell function, the death of Jurkat cells was decreased in the co-culture model of PC-3 cells and Jurkat cells when the PC-3 cells were transfected with shCAR (Fig. 7C). To further evaluate the T cell cytotoxic function, we detected the secretion of IFN-γ, IL-2, and TNF-α in the co-culture system by using RT-qPCR and ELISA assays, and found that knockdown of CARHSP1 resulted in higher levels of these immune-promoting cytokines secreted by Jurkat cells, especially IFN-γ (Fig. 7D and E). In brief, these results indicated that CARHSP1 promoted immunosuppression. To rule out cytotoxicity effects in the co-culture model, we treated Jurkat cells alone with IL-17 A, and flow cytometry analysis for apoptosis demonstrated that IL-17 A has no killing effect on Jurkat cells (Fig. 7F). Furthermore, the results in the co-culture model showed that IL-17 A stimulation reversed the promotional effects of CARHSP1 knockdown on T cell-mediated cancer cell killing, indicating that CARHSP1 partially depended on IL-17 A/IL-17RA to participate in the suppression of the anti-tumor immune microenvironment (Fig. 7G).

Taken together, these findings demonstrate that CARHSP1 facilitates the immune evasion of PCa cells through modulation of the IL-17RA/NF-κB/PD-L1 signaling.

## Discussion

Cold shock domain (CSD) proteins are characterized by the presence of one or more evolutionarily conserved cold shock domains, which can be found in over 6,000 proteins from prokaryotes to vertebrates, except yeast [24, 25]. The cold shock domain protein family contains many nucleic acid binding proteins, which exert pleiotropic functions in cells via their ability to bind single-stranded RNA and/or DNA, thus allowing them to serve as transcriptional as well as translational regulators [26]. Calcium-regulated heat-stable protein 1 (CARHSP1) belongs to the CSD protein family, and other cold shock domain proteins are also found in humans: the Y-box subgroup [27], abnormal cell lineage protein 28 homolog A, B (LIN28A and B) [28], upstream of N-Ras (UNR) [29], and PIPPin [30]. Recent findings indicate that the involvement of CARHSP1 in the regulation of RNA stability plays a crucial role in tumor progression [7, 8]. However, CARHSP1 has rarely been studied in PCa. In this study, IL-17RA was firstly identified as a direct target of RNA stability regulation by CARHSP1. Mechanistically, the RNA-binding protein CARHSP1 identified and selectively bound to the mRNA of IL-17RA, resulting in the increased expression of both IL-17RA mRNA and protein. Downregulating expression of CARHSP1 shortened the half-life of IL-17RA mRNA and reduced its expression. However, the specific binding region was not identified in this study and future studies are needed to clarify the specific binding elements or regions of CARHSP1 and IL-17RA. The binding sequence was not ARE.

Interleukin-17 (IL-17), which plays an important role in inflammation, immunity, and autoimmunity, has been found increased in several tumors, including prostate cancer [20, 31, 32]. IL-17RA, a receptor for IL-17 A and IL-17 F, is the founding member of the IL-17 receptor (IL-17R) family and seems to function as a co-receptor with at least 2 other members of the IL-17-ligand family. IL-17RA is expressed ubiquitously and can be dynamically regulated [33, 34], which could be biologically significant because IL-17 A-induced signaling strength correlates with cell surface expression levels of IL-17RA [35]. With ever increasing evidence of the vital role of IL-17 signaling pathway in pathogenic inflammation conditions, it is inevitable that attention is also now turning towards identifying candidate drugs targeting IL-17 signaling pathway that can serve as a potential anti-inflammatory strategy for the treatment of inflammatory autoimmune diseases. There are three anti-IL-17 monoclonal antibody agents which have approved by the US Food and Drug Administration for the treatment of moderate-to-severe plaque psoriasis: secukinumab [36] and ixekizumab [37] (IL-17 A antagonists), and brodalumab [38] (IL-17RA antagonist). A recent study has demonstrated that Gingerenone A, a phenolic compound isolated from Zingiber officinale, is able to improve intestinal mucosal inflammation and restore intestinal barrier homeostasis by inhibiting IL-17RA signaling to ameliorate ulcerative colitis (UC) [39]. However, the role of IL-17 signaling in carcinogenesis has been quite controversial. Both beneficial and detrimental effects of IL-17 signaling occurred in context-dependent and tumor-system-dependent manners [40]. Many independent groups have demonstrated that IL-17 promotes development of prostate cancer in animal models [21, 41]. Moreover, a significant correlation has also been found between Th17 cell differentiation and prostate cancer progression in clinical settings [42]. IL-17 A is the vital cytokine secreted by Th17 cells [43], and several studies have further confirmed that Th17-IL-17 pathway could play an important role in development of primary prostate cancer and lymph node metastasis [44–46]. However, it’s notable that the high IL-17 level secreted by Th17 cells could not only promote development of prostate cancer cells, but also enhance the survival and functionality of CD8^+^ T cells to combat cancer cells [47]. These are consistent with the relevant phenotypes obtained in our experiments. We reduced the expression of IL-17RA by knocking down CARHSP1, which significantly weakens the capacity of proliferation, migration, invasion, and immune evasion of PCa cells. Moreover, rescue assays further confirmed the cancer-promoting role of IL-17 signaling in PCa by using recombinant human IL-17 A stimulations.

IL-17 binds to IL-17R, activating hallmark pathways associated with cancer [48]. For example, it has been reported that nuclear factor-κB (NF-κB) pathway could be activated by IL-17 A signaling. The IL-17RA/IL-17RC receptor complex, as the main active place of IL-17, recruits and activates NF-κB activator 1 (Act1) [49]. Act1 is an E3 ubiquitin ligase that activates tumor necrosis factor receptor-associated factor 6 (TRAF6) through lysine-63-linked ubiquitination. The polyubiquitinated TRAF6 then activates transforming growth factor-β-activated kinase 1 (TAK1) and subsequently IκB kinase (IKK) complex, finally resulting in activation of NF-κB pathway that induces transcription of a variety of chemokines and cytokines, such as C-X-C motif ligand 1 (CXCL1), C-C motif ligand 20 (CCL20), IL-1β and IL-6 [50, 51]. This, in turn, increases Th17 differentiation. A recent study has also shown that increased IL-6 production promotes naive T-cell differentiation to Th17 [52]. As Th17 cells produce IL-17, its lineage expansion contributes to enhance IL-17 secretion. Thus, it is interesting to note that IL-17 secretion is regulated by a positive feedback mechanism and the interaction between IL-17 and IL-6 could amplifies the production of both cytokines [53]. It has been widely documented that IL-6 mediated activation of STAT3 signaling further triggers the transcription of various downstream target genes, including MMPs, Cyclin family members, and EMT makers, enhancing tumor cell survival, proliferation and invasion [54]. Consistently, in the present study, we found that NF-κB pathway and JAK/STAT3 pathway were down-regulated by CARHSP1 knockdown and rescued by IL-17 A stimulation, indicating that the downstream pathways of IL-17RA were affected by CARHSP1 and supporting that CARHSP1 promote cancer progression through regulation on IL-17RA.

Up to date, there is enough evidence that an inflammatory microenvironment plays a critical role in tumor initiation, growth, and development [55, 56]. 15−20% of cancers are linked to chronic infections, and most solid tumors trigger an intrinsic inflammatory response that builds up a protumorigenic microenvironment. Almost all surgical prostate specimens contain evidence of inflammation [57, 58]. As stated above, IL-17 signaling triggered activation of STAT3 pathway and NF-κB pathway consequently promotes the release of a variety of proinflammatory factors and chemokines from tumor cells, which can contribute to maintain the proinflammatory reaction locally [59]. PD-1/PD-L1 signaling axis has been demonstrated to play a role in regulating tumor microenvironment of prostate cancer, and Gevensleben et al. found that 52.2% of 209 primary prostate cancer samples expressed moderate to high PD-L1 levels and PD-L1 positivity was prognostic for biochemical recurrence [60]. However, how PD-L1 expression is regulated in prostate cancer remains elusive. PD-L1 is known to be highly expressed under inflammatory conditions, such as in the presence of IFN-γ and IL-6 [61, 62]. Therefore, it’s naturally for us to speculate that factors related to IL‐17/Th17 and PD‐L1 may be involved in the immune response caused by inflammation. In fact, IL-17 has been reported to positively associate with PD-L1 upregulation in cancers [53]. As shown in Fig. 6, when we knocked down CARHSP1, the PD-L1 expression was significantly decreased via the regulation of IL-17RA, resulting in less cytotoxic T cell exhaustion and more PCa cell death in a co-culture model in *vitro*. Overall, our results demonstrate that as a regulator of IL-17RA and PD-L1, CARHSP1 plays an important role in the modulation of tumor microenvironment in PCa.

There are some highlights of this study. As far as we know, the roles and functions of CARHSP1 in PCa in vitro and in vivo have not been reported yet. On the one hand, bioinformatics analysis, gain- and loss-of-function assays in vitro, and different in vivo study models were integrated in our study, making our results more credible. On the other hand, we uncovered that IL-17RA, a direct target of CARHSP1, serves as a mediator of CARHSP1 by regulating the activation of STAT3 and NF-κB signaling pathways and thereby affecting PCa progression. However, this study still has some limitations. CARHSP1 expression in human PCa tissues have not been detected due to the lack of appropriate antibody for IHC assay. This kind of investigation will be performed in the future. Additionally, compared with the subcutaneous transplantation tumor model, the orthotopic transplantation model can better simulate the tumor microenvironment and the whole process of tumor growth and metastasis, which should also be performed in the future.

**Fig. 8:**
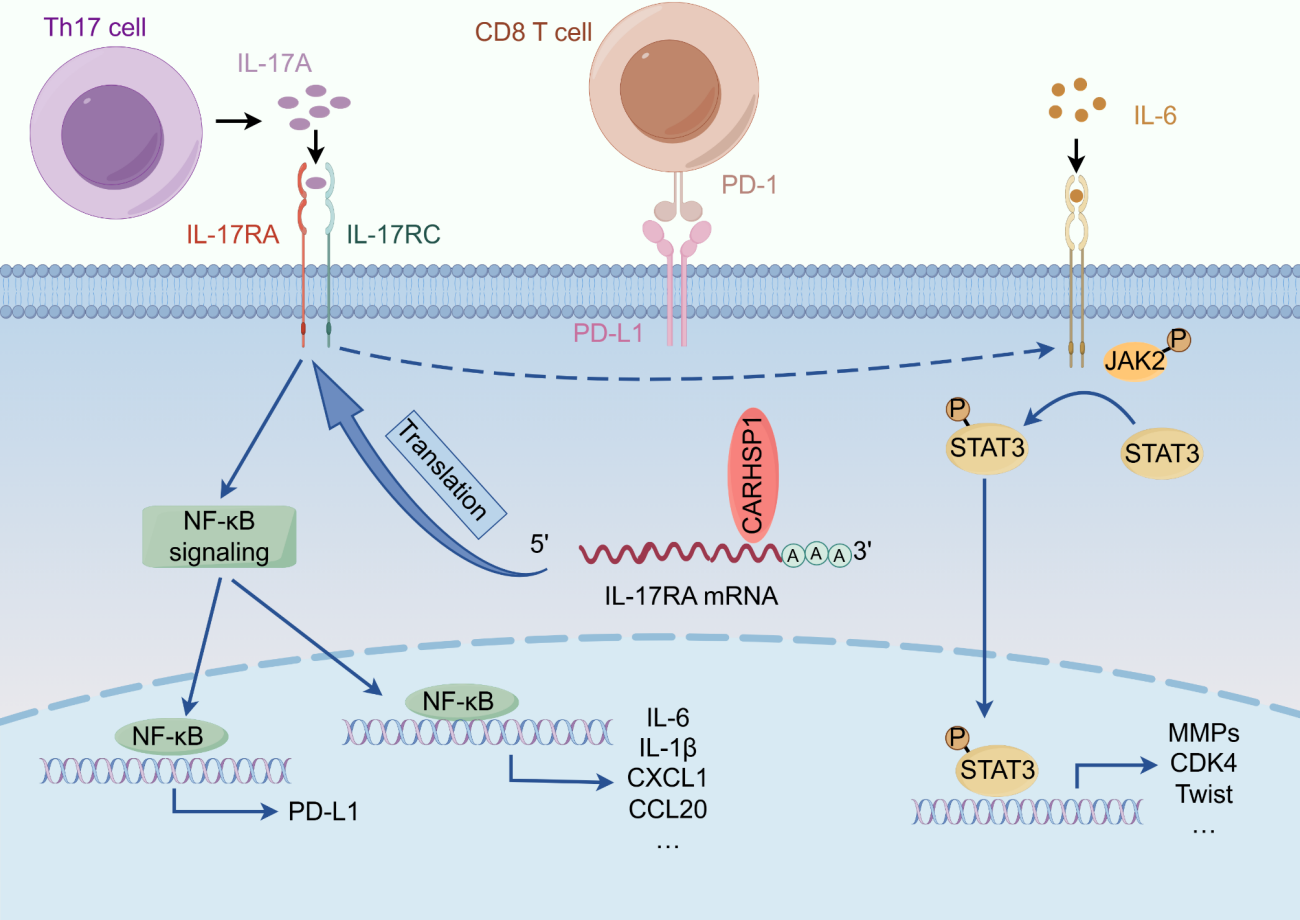
An illustration of how CARHSP1 facilitates tumor growth, metastasis and immune escape by enhancing IL-17RA mRNA stabilization in prostate cancer. The figure was created using Figdraw

In the present study, we found that upregulation of CARHSP1 could significantly enhance the capacity of proliferation, migration, invasion, and immune evasion of PCa cells. Mechanistically, CARHSP1 directly bound to the 3’-UTR of IL-17RA mRNA as an RBP and enhanced the stability of IL-17RA mRNA, resulting in elevated expression of IL-17RA. IL-17/IL-17RA subsequently activated NF-κB pathway and STAT3 pathway, triggering the transcription of various downstream target genes, including PD-L1, Cyclin family members, MMPs, and EMT makers (Fig. 8), which may lead to immune evasion, tumor growth, and tumor invasion of PCa. Thus, these results demonstrate that CARHSP1 promotes the progression of PCa through modulating the expression of IL-17RA and activating its downstream pathway, and suggest that CARHSP1/IL-17RA axis could be a potential novel therapeutic target for PCa.

## Conclusions

In conclusion, our results demonstrated that the increased expression of CARHSP1 in PCa is correlated with advanced clinical characteristics and unfavorable prognosis, and CARHSP1 may promote the progression of PCa through enhancing the mRNA stability of IL-17RA and activating its downstream pathways. This is the first comprehensive study on CARHSP1 in PCa and a new target of CARHSP1, IL-17RA, is identified for the first time. These results suggest that CARHSP1 is an important regulator of tumor microenvironment in PCa, and CARHSP1/IL-17RA axis could be potential novel therapeutic targets for PCa.

## Electronic supplementary material

Below is the link to the electronic supplementary material.

Supplementary Material 1

Supplementary Material 2

## Data Availability

The data generated in this study are available upon request from the corresponding author.
