# Supplementary material for: The RNA binding protein CARHSP1 facilitates tumor growth, metastasis and immune escape by enhancing IL-17RA mRNA stabilization in prostate cancer
Source: Cell Biosci. 2025 Mar 7;15:33. doi: 10.1186/s13578-025-01371-4 (PMC11889941; doi:10.1186/s13578-025-01371-4)
Supplement: Supplementary file 1 — Supplementary Material 1 [file 13578_2025_1371_MOESM1_ESM.docx]

**Supplementary materials**

**Supplementary Table S1. Primers for RT-qPCR**

| Gene | Forward primer sequence | Reverse primer sequence |
| --- | --- | --- |
| *18S* | GCAATTATTCCCCATGAACG | GGCCTCACTAAACCATCCAA |
| *CARHSP1* | CCCCGTCTACAAAGGAGTCTG | GGGTGGGATGGAGCACATTT |
| *MMP2* | TACAGGATCATTGGCTACACACC | GGTCACATCGCTCCAGACT |
| *MMP9* | AGACCTGGGCAGATTCCAAAC | CGGCAAGTCTTCCGAGTAGT |
| *IL-17RA* | GACACTCCGCGACTGTTTC | GCCCGTGATGAACCAGTACAC |
| *IL-17RB* | GGCTGCCTAGACCACATAATG | GCTGTGTTGGATAAGAGCCAT |
| *IL-17RC* | GATGGTGACAACGTGCATCTG | CAAGGTAATGATCTGCGGTCC |
| *GAPDH* | GATTCCACCCATGGCAAATTC | CTGGAAGATGGTGATGGGATT |
| *IFNγ* | TCGGTAACTGACTTGAATGTCCA | TCGCTTCCCTGTTTTAGCTGC |
| *IL2* | AACTCCTGTCTTGCATTGCAC | GCTCCAGTTGTAGCTGTGTTT |
| *TNFα* | CCTCTCTCTAATCAGCCCTCTG | GAGGACCTGGGAGTAGATGAG |
| *Carhsp1* | GCTGGAGGAGTAGGACGTGT | ACAGAAGTCTGATGCGTGGG |

**Supplementary Table S2. RNA sequence used in RNA pull down experiments**

| RNA | Sequence | AREs Region |
| --- | --- | --- |
| *RNA1* | CAGCAUUUAUUGUGCACCUA | 2861-2880 |
| *RNA2* | GUUCAUUUAUAGGAAGAGAG | 3989-4008 |
| *RNA3* | GUCCAUUUAGGUUGAUGAAA | 7113-7132 |
| *RNA4* | UGUGAAAUGUAGGCUUUAAAAUGUAAAUGUCUGGAUUUUAAUCCCAGGCAUCCCUCCUAA | 2692-2751 |
| *NS RNA* | UCUACCUCUAGGAGGUGUGA |  |


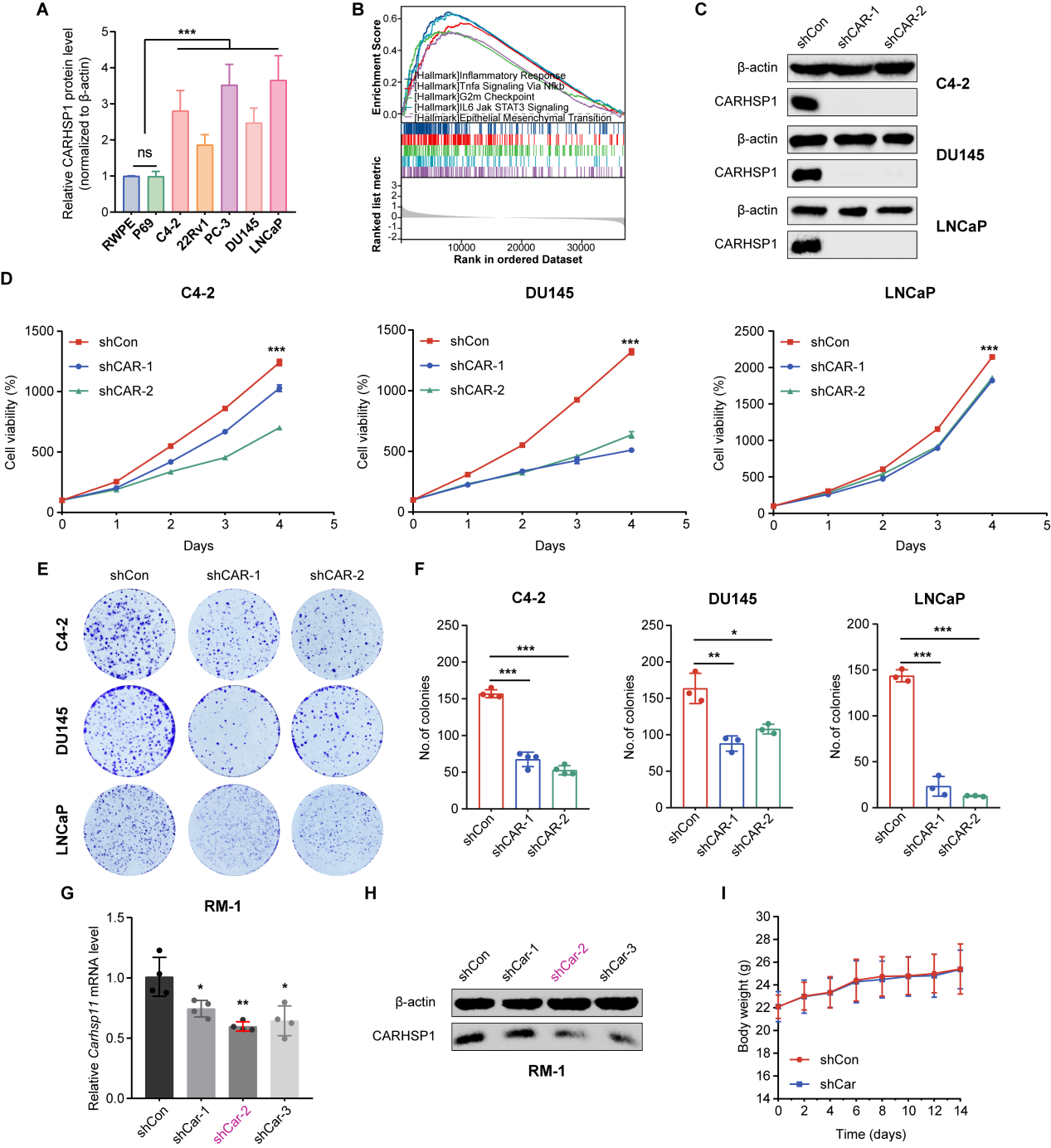


**Supplementary Fig. 1 CARHSP1 promotes prostate cancer proliferation.** (A) Quantitative analysis of the relative expression of CARHSP1 in different cell lines by Western blotting (normalized to β-actin). (n = 3, mean ± SD). (B) GSEA results based on the TCGA PCa cohort identifying several signaling pathways that were upregulated and responded to high CARHSP1 expression. (C) Western blotting analysis of CARHSP1 protein levels in C4-2, DU145, and LNCaP cells transfected with CARHSP1 shRNAs (shCAR) or control (shCon). β-actin was used as the internal loading control. (D) Cell viability in C4-2, DU145, and LNCaP cells transfected with shCAR or shCon detected by MTT assay. (n = 3, mean ± SD). (E, F) Representative images of the colony formation assay in C4-2, DU145, and LNCaP cells transfected with shCAR or shCon and quantification analyses of the colony number. (n = 3, mean ± SD). (G, H) CARHSP1 levels in RM-1 cells transfected with *Carhsp1* shRNAs (shCar) or control (shCon). (I) The body weight changes of C57BL/6 mice in the RM-1/shCon group and RM-1/shCar group. **p* < 0.05; ***p* < 0.01; ****p* < 0.001.


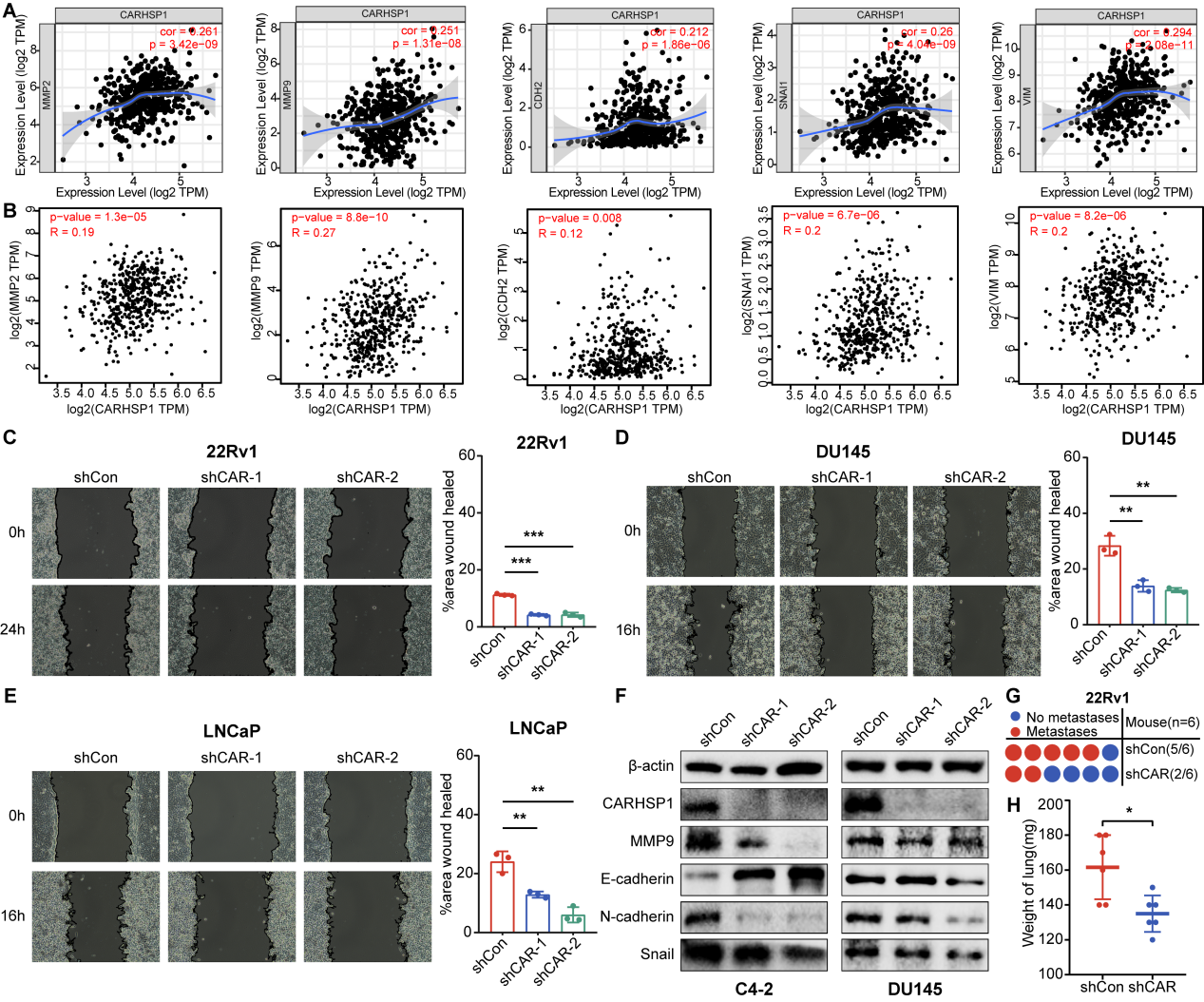


**Supplementary Fig. 2 CARHSP1 promotes prostate cancer metastasis.** (A, B) Correlation analysis between mRNA expression levels of CARHSP1 and metastasis and EMT biomarkers in PCa based on the TCGA cohort using TIMER (A) and GEPIA (B) database. (C-E) Representative images of wound healing assays in 22Rv1, DU145, and LNCaP cells transfected with shCAR or shCon and quantitative data (n = 3, mean ± SD). (F) Western blotting analysis of metastasis and EMT biomarkers levels after CARHSP1 knockdown in C4-2 and DU145 cells. β-actin was used as a loading control. (G) Quantification of lung metastases in mice bearing either 22Rv1/shCon tumors or 22RV1/shCAR tumors. (H) Lung weight of the mice 22Rv1 cell lung metastasis. **p* < 0.05; ***p* < 0.01; ****p* < 0.001.


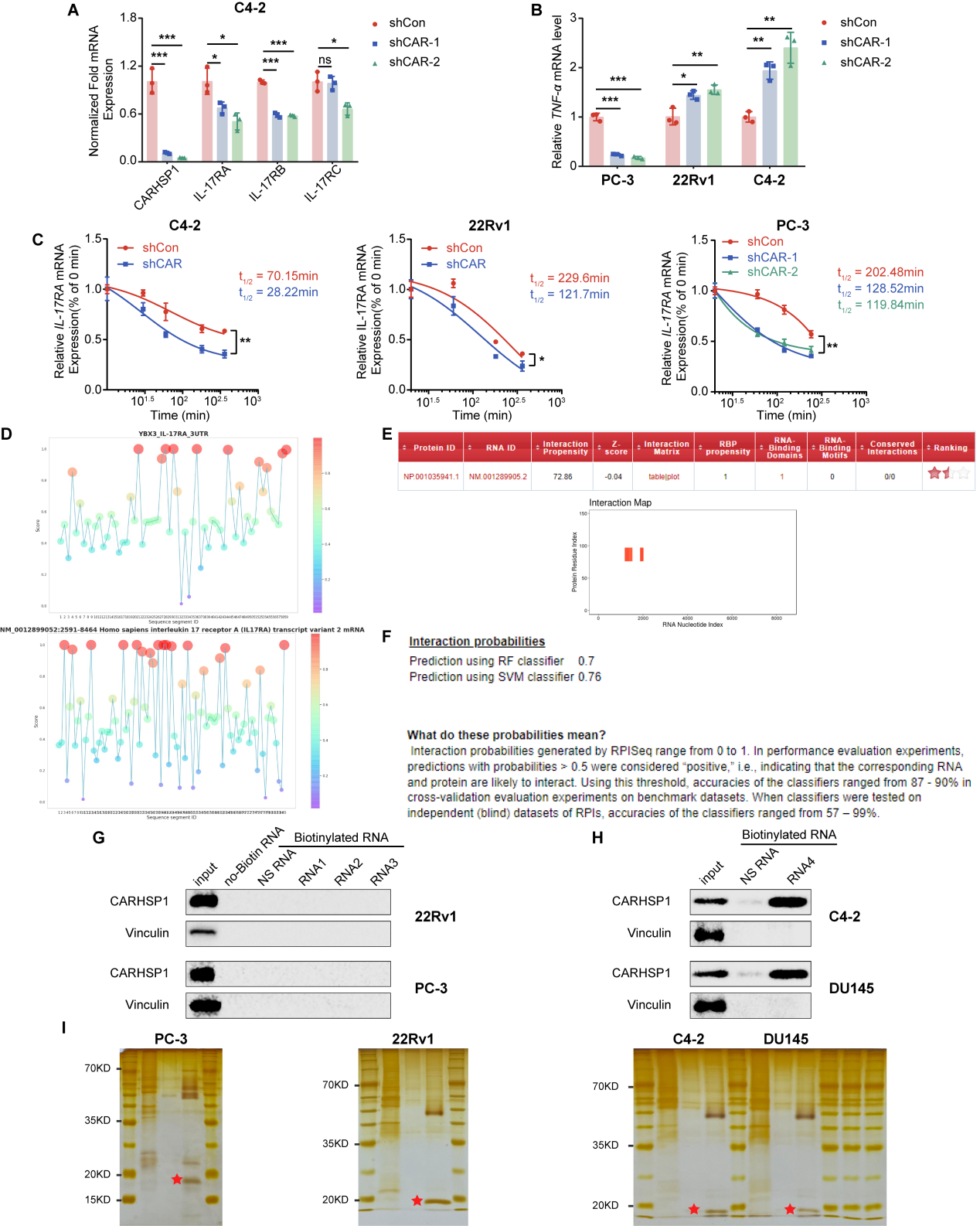


**Supplementary Fig. 3** **IL-17RA is a direct target of CARHSP1.** (A) Effect of CARHSP1 knockdown on the expression of IL-17 receptor family members *IL-17RA*, *IL-17RB* and *IL-17RC* in C4-2 cells, as detected by RT-qPCR. 18S was used as an internal loading control. (B) Effect of CARHSP1 knockdown on the expression of *TNF-α* mRNA in PC-3, 22Rv1, and C4-2 cells, as detected by RT-qPCR. 18S was used as an internal loading control. (C) Measurement of *IL-17RA* mRNA stability by RT-qPCR after C4-2, 22Rv1, and PC-3 cells transfected with shCAR or shCon were exposed to Actinomycin D (5 μg/mL). The data are expressed as the percentage of mRNA molecules before the Actinomycin D treatment (n = 3, mean ± SD). (D) YBX3 was potential to interact with *IL-17RA* 3’-UTR via RBPsuite database. (E) CARHSP1 binding sites in *IL-17RA* mRNA predicted by catRAPID. (F) CARHSP1 may have potential to interact with *IL-17RA* 3’-UTR (RPISeq database). (G) Analysis of the interaction of CARHSP1 with the *IL-17RA* 3’-UTR (*RNA1/2/3*) by RNA pull-down assays followed by western blot analysis in 22Rv1 and PC-3 cells. Vinculin was used as a loading control. (H, I) Analysis of the interaction of CARHSP1 with the *IL-17RA* 3’-UTR (*RNA4*) by RNA pull-down assays followed by western blotting analysis in C4-2 and DU145 cells and silver staining in PC-3, 22Rv1, C4-2 and DU145 cells. Vinculin was used as a loading control. **p* < 0.05; ***p* < 0.01; ****p* < 0.001; ns, not significant.


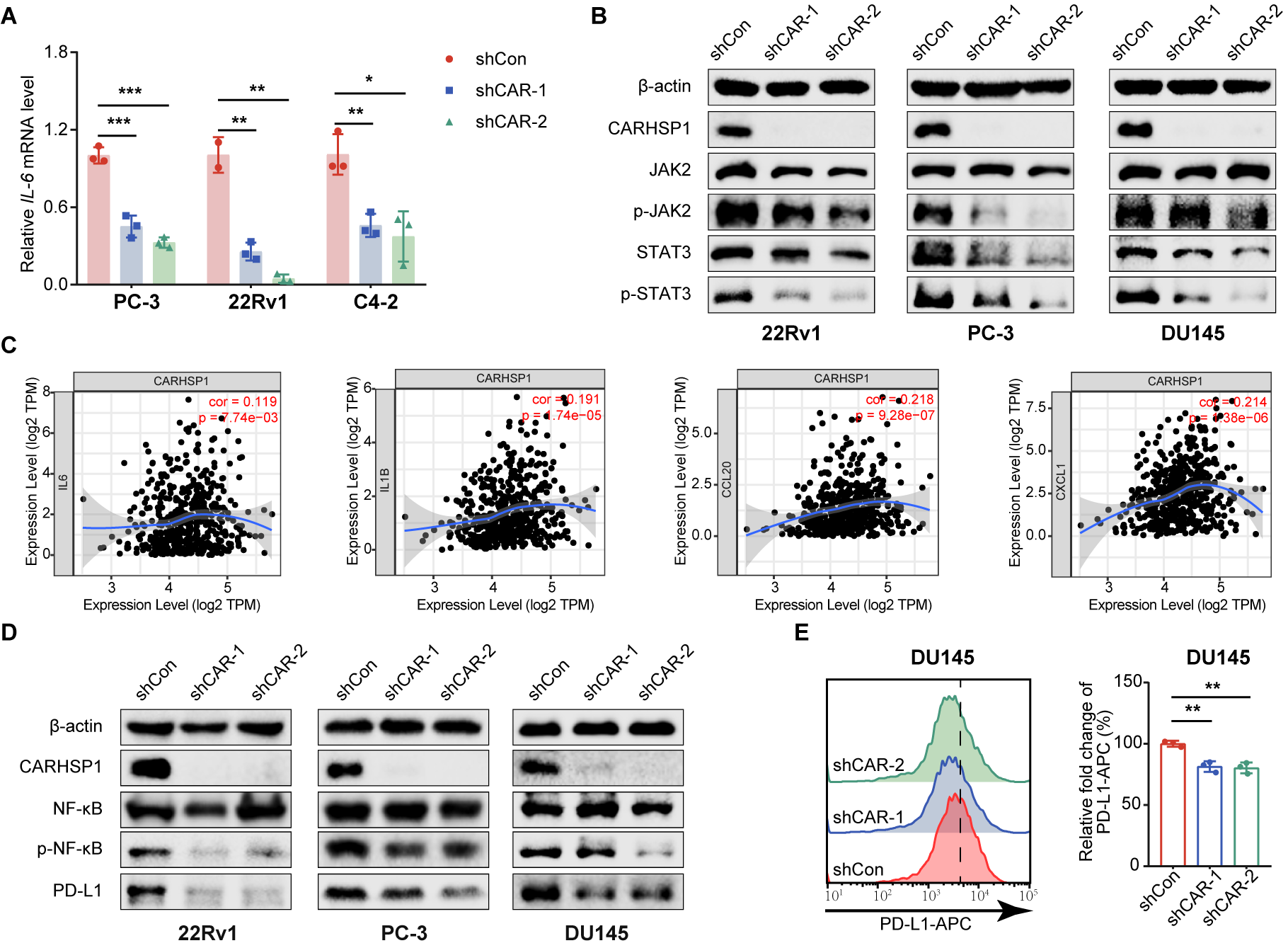


**Supplementary Fig. 4 CARHSP1 facilitates tumor progression via the IL-17RA-dependent activation of STAT3 pathway and NF-κB pathway in PCa.** (A) Effect of CARHSP1 knockdown on the expression of *IL-6* mRNA in PC-3, 22Rv1, and C4-2 cells, as detected by RT-qPCR. 18S was used as an internal loading control. (B) Western blotting analysis of CARHSP1, JAK2, p-JAK2, STAT3, and p-STAT3 in 22Rv1, PC-3, and DU145 cells transfected with shCAR or shCon. β-actin was used as a loading control. (C) Correlation analysis between mRNA expression levels of CARHSP1 and chemokines and cytokines induced by NF-κB pathway in PCa based on the TCGA cohort using TIMER database. (D) Western blotting analysis of CARHSP1, NF-κB, p-NF-κB, and PD-L1 in 22Rv1, PC-3, and DU145 cells transfected with shCAR or shCon. β-actin was used as a loading control. (E) Analysis of cell surface PD-L1 protein using flow cytometry in DU145 cells with stable depletion of CARHSP1 (n = 3, mean ± SD). ** *p* < 0.01 and *** *p* < 0.001.
