# Supplementary material for: The RNA binding protein CARHSP1 facilitates tumor growth, metastasis and immune escape by enhancing IL-17RA mRNA stabilization in prostate cancer
Source: Cell Biosci. 2025 Mar 7;15:33. doi: 10.1186/s13578-025-01371-4 (PMC11889941; doi:10.1186/s13578-025-01371-4)

**Supporting information**

**The RNA binding protein CARHSP1 facilitates tumor growth, metastasis and immune escape by enhancing IL-17RA mRNA stabilization in prostate cancer**

**Yi-Fan Jiang^1^*, Ya-nan Wang^1^*, Kai-Hua Xue^1^*, Jian-Bin Ma^1^, Shan Xu^1,2,3^, Ke Wang^1,2,3^, Peng Guo^1,2,3^**

**This file shows the whole un-cropped images of the original western blots.**

**Figure 1**

**(E)**


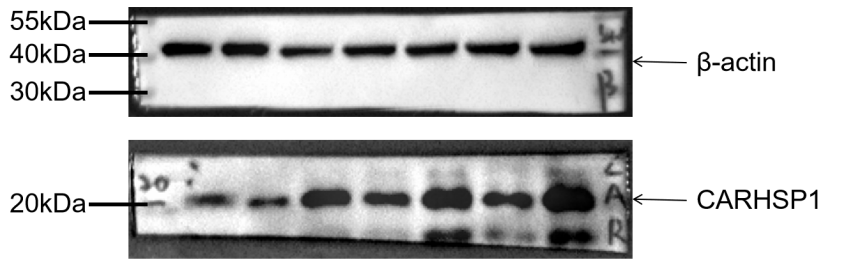


**Figure 2**

**(A)**


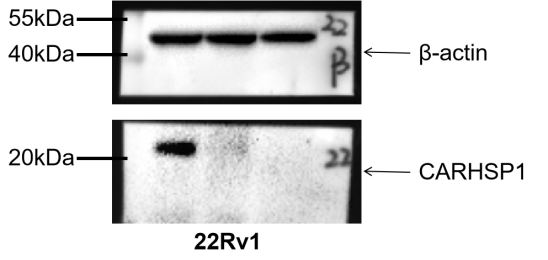


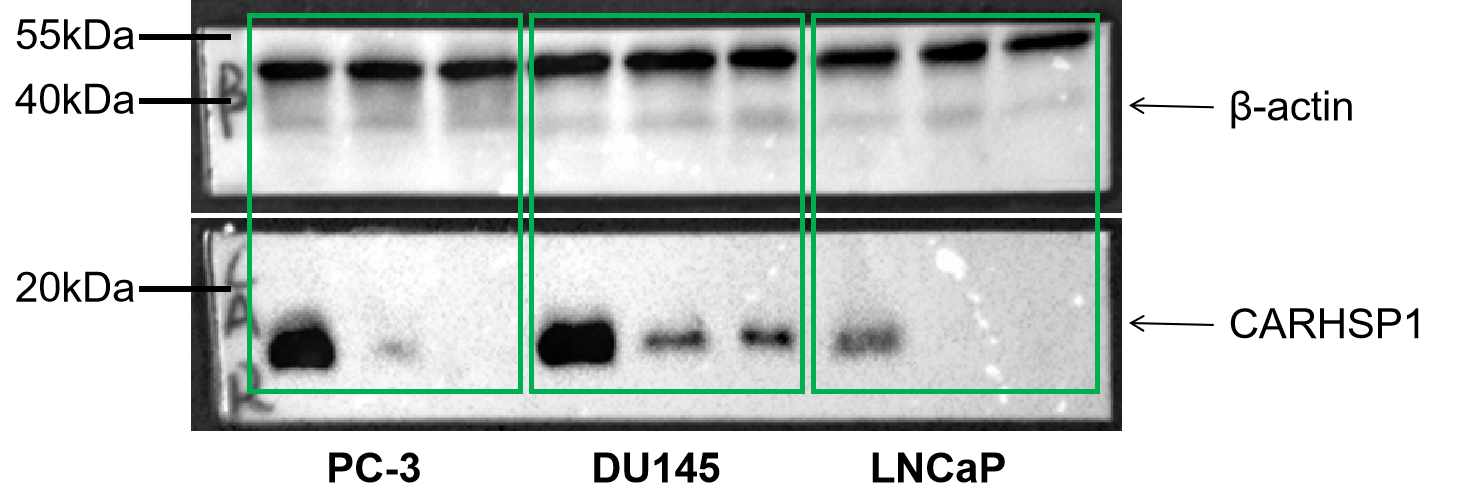


**(E)**


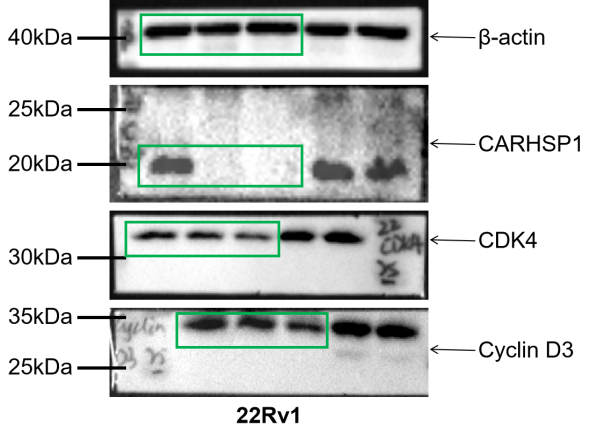


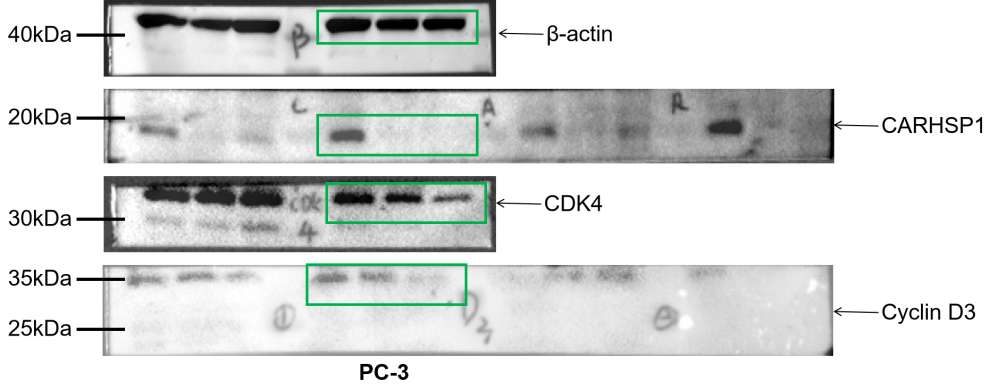


**Figure 3**

**(I)**


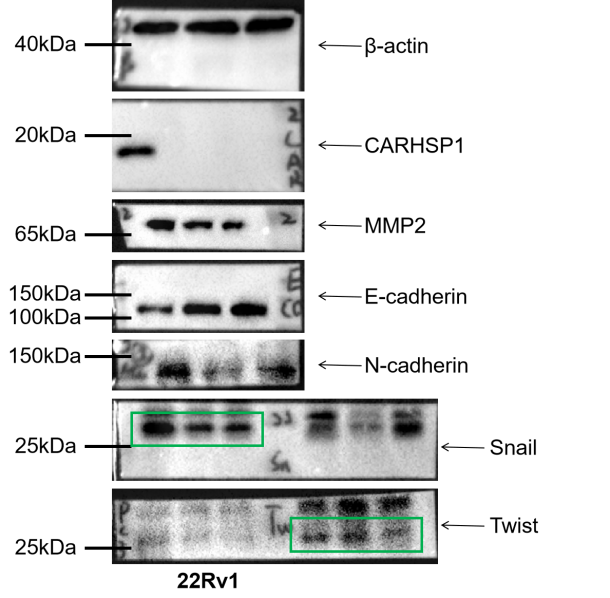


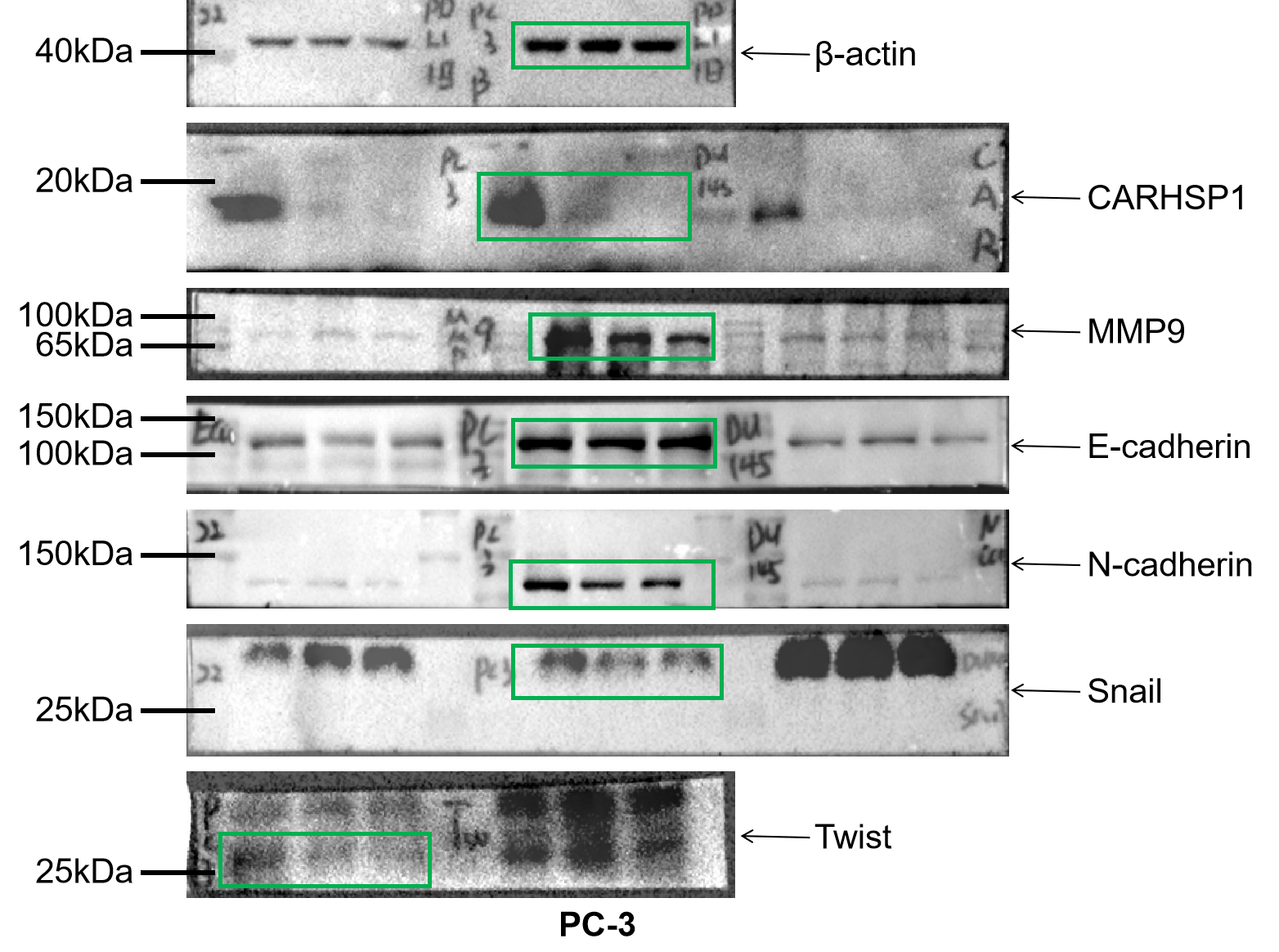


**Figure 4**

**(F)**


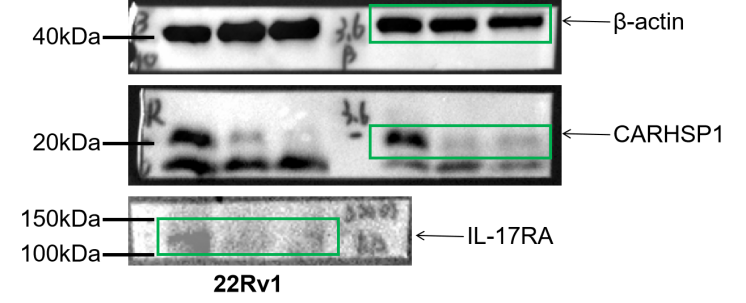


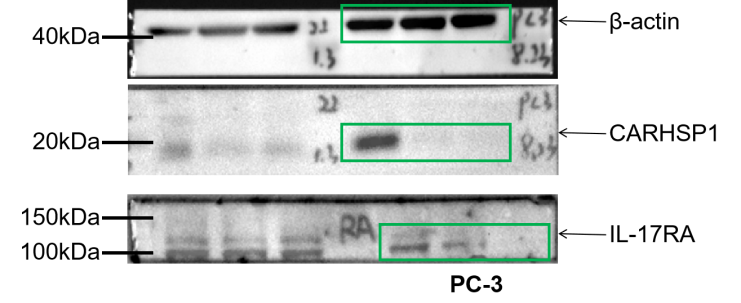


**(J)**


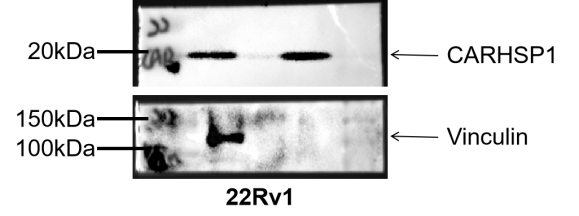


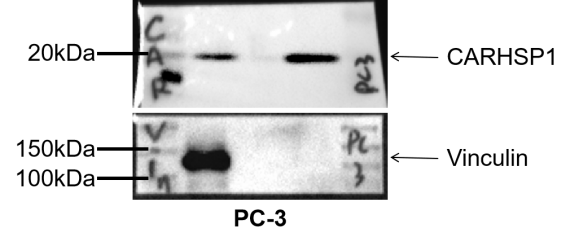


**Figure 5**

**(B)**


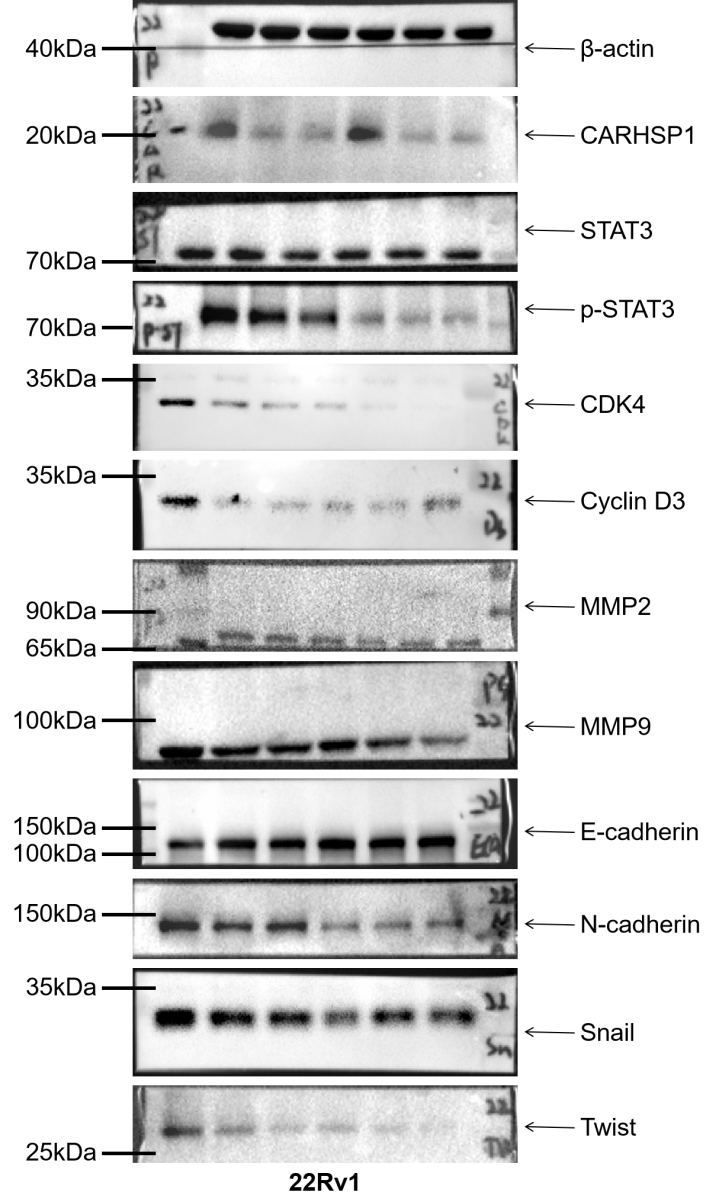


**Figure 5**

**(B)**


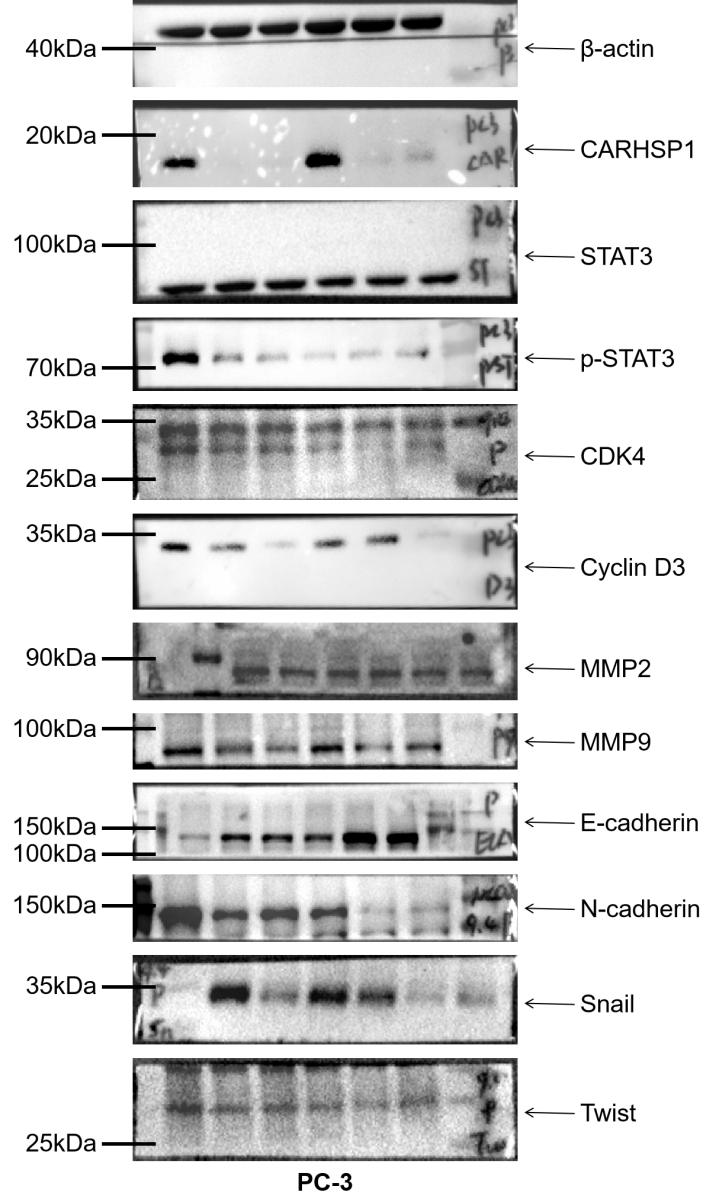


**Figure 5**

**(D)**


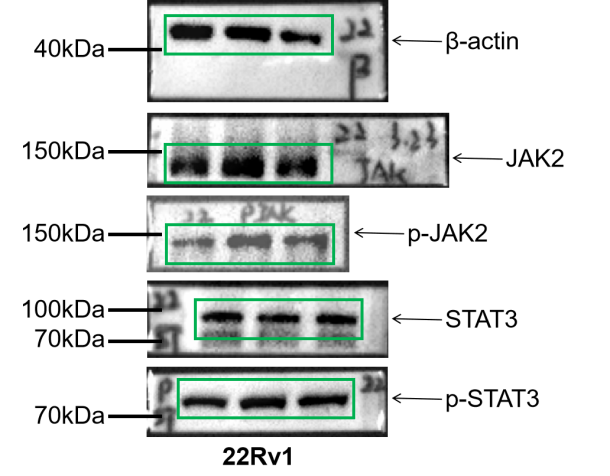


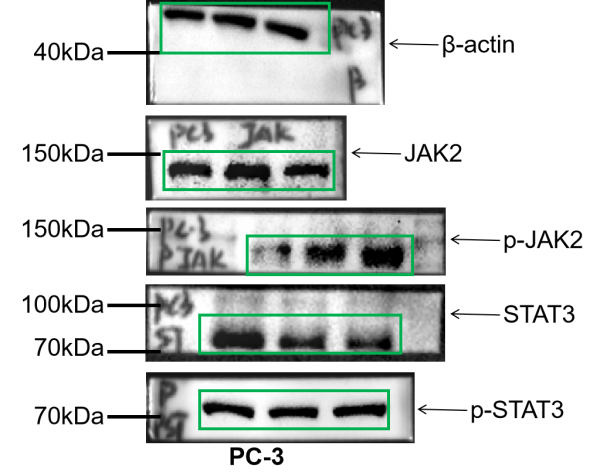


**Figure 6**

**(E)**


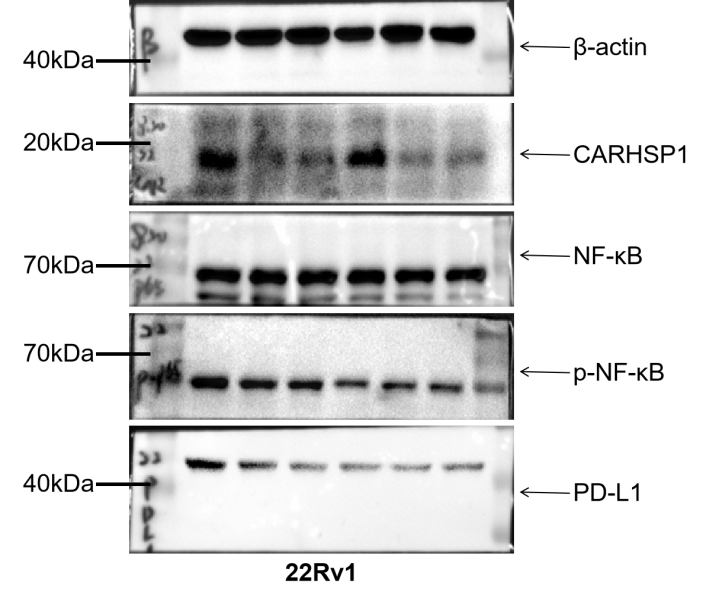


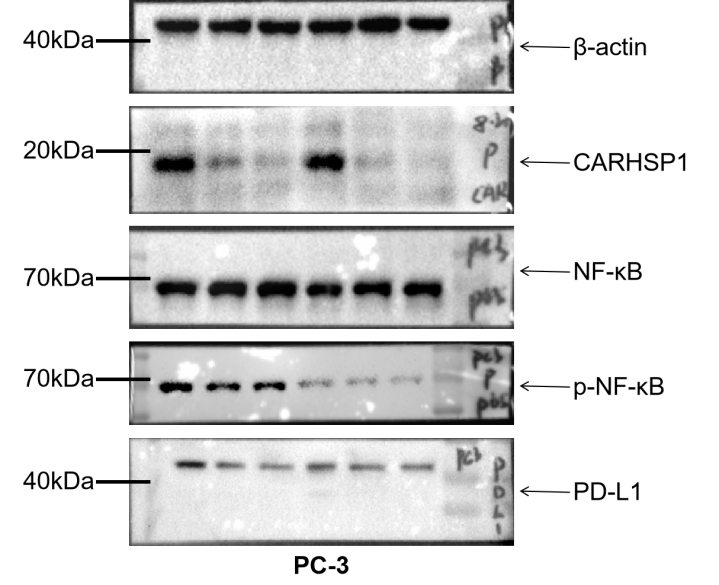


**Figure 6**

**(H)**


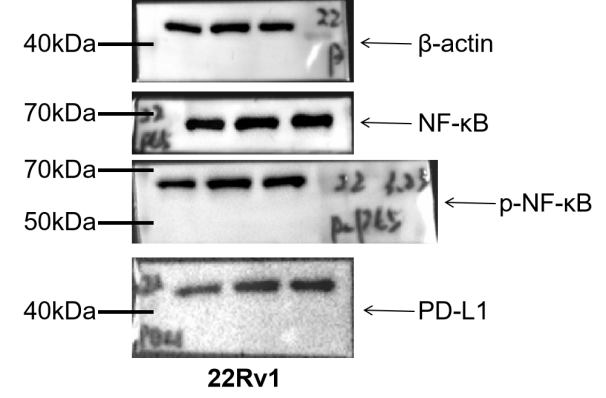


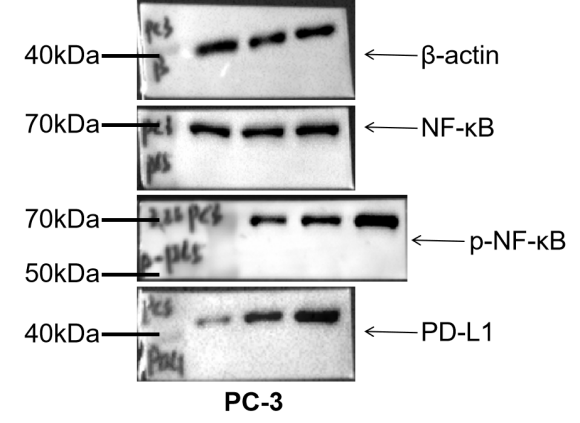


**Figure S1**

**(B)**


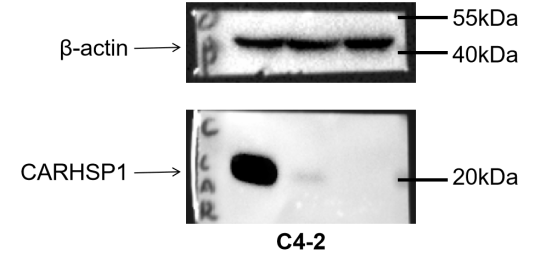


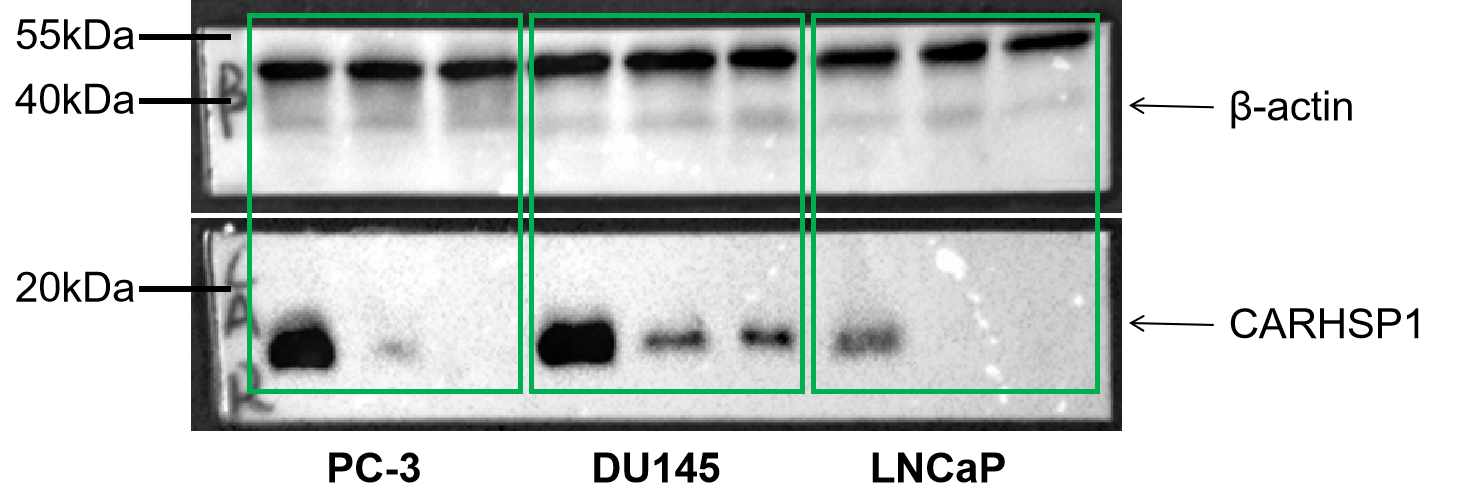


**(G)**


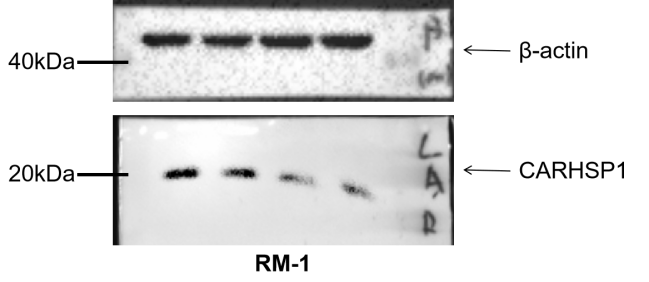


**Figure S2**

**(F)**

**
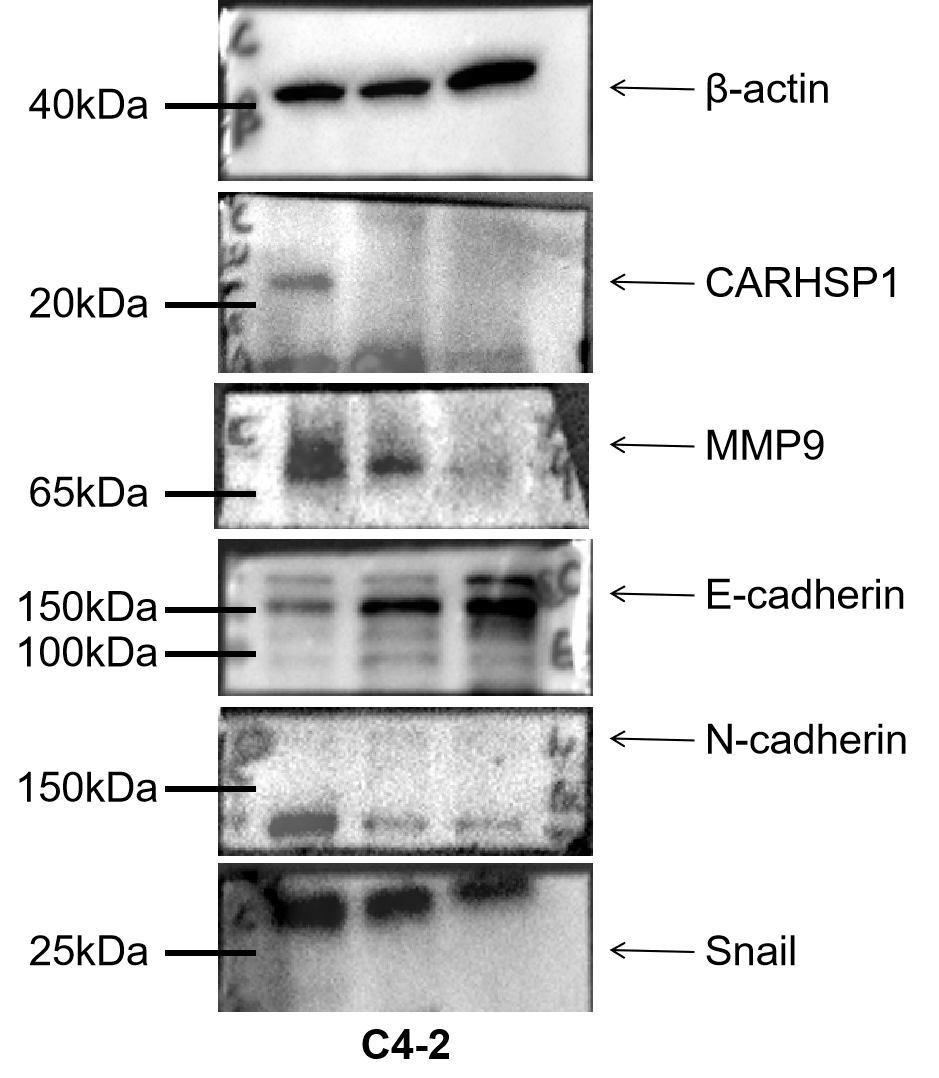
**

**
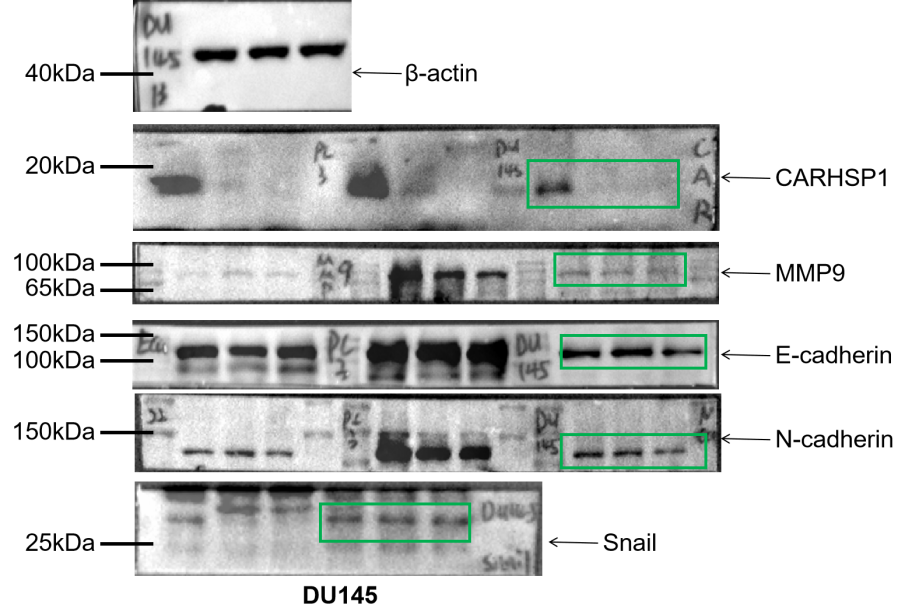
**

**Figure S3**

**(G)**

**
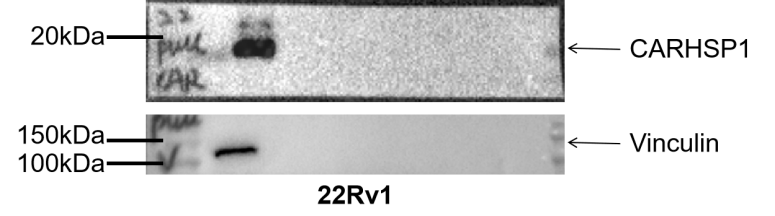
**

**
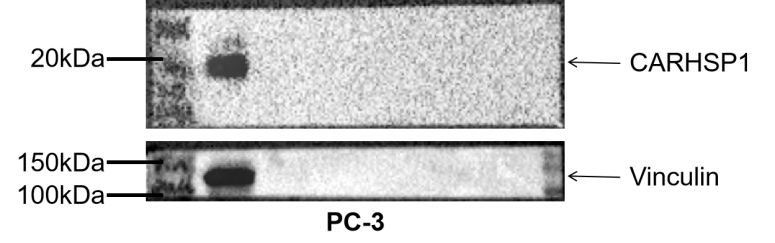
**

**(H)**

**
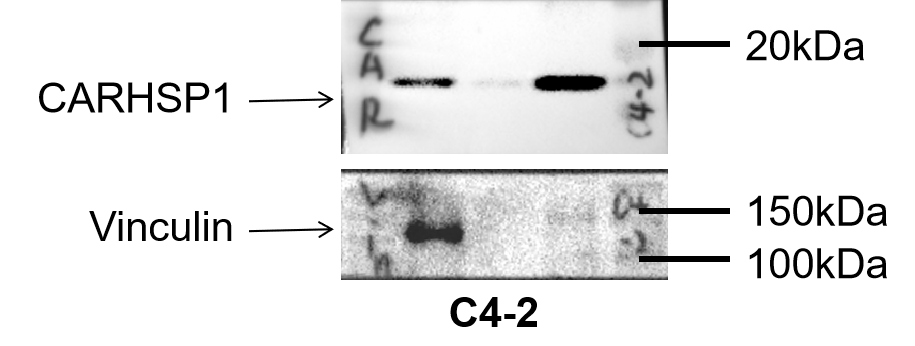
**

**
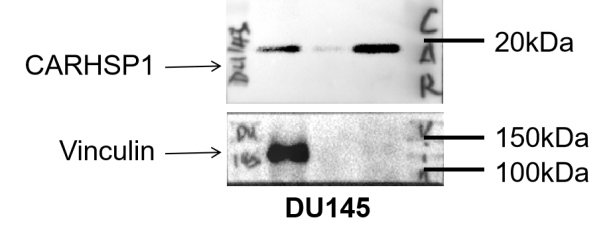
**

**Figure S4**

**(B)**


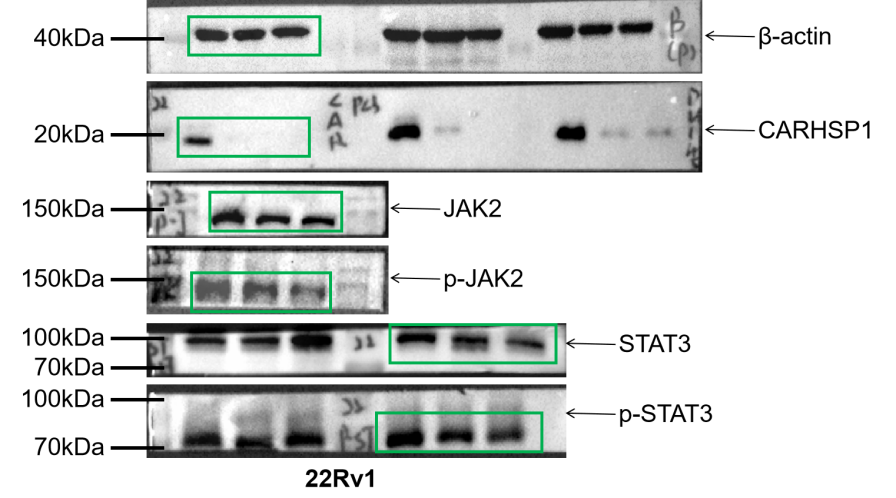


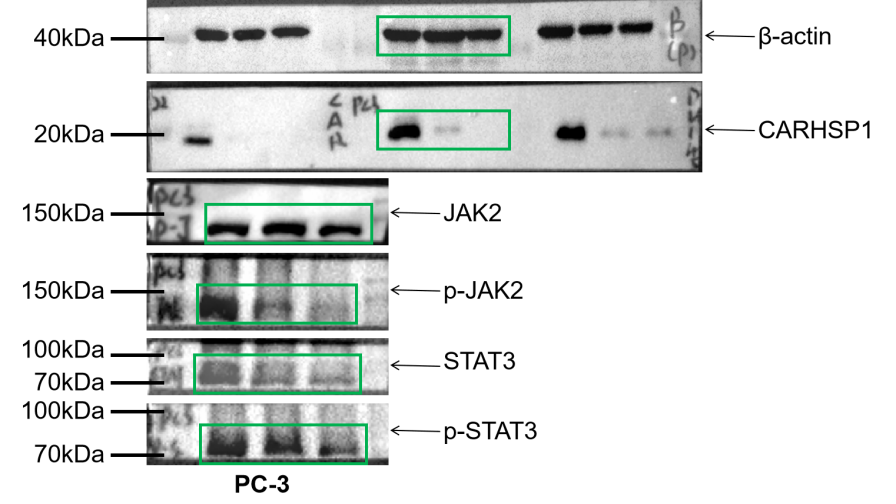


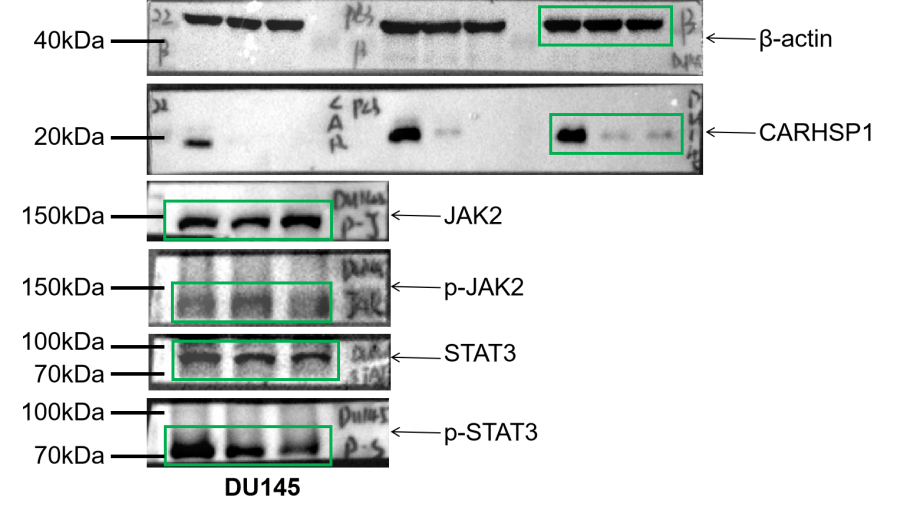


**Figure S4**

**(D)**


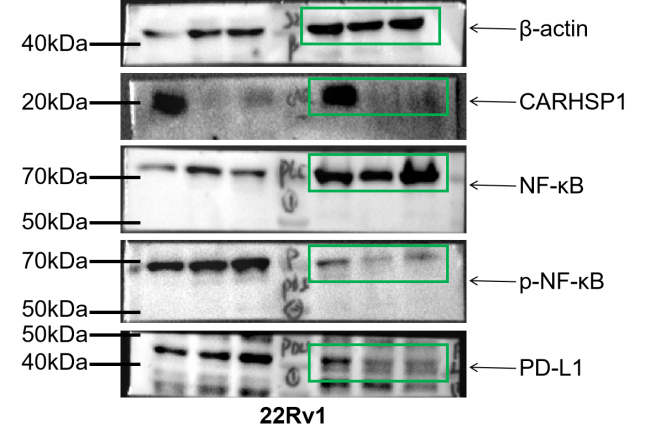


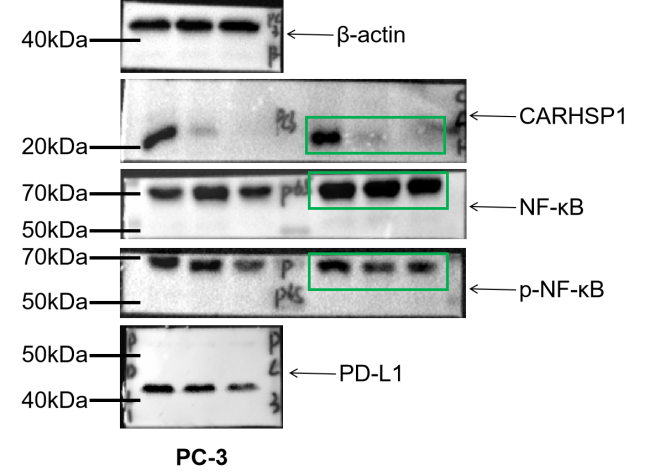


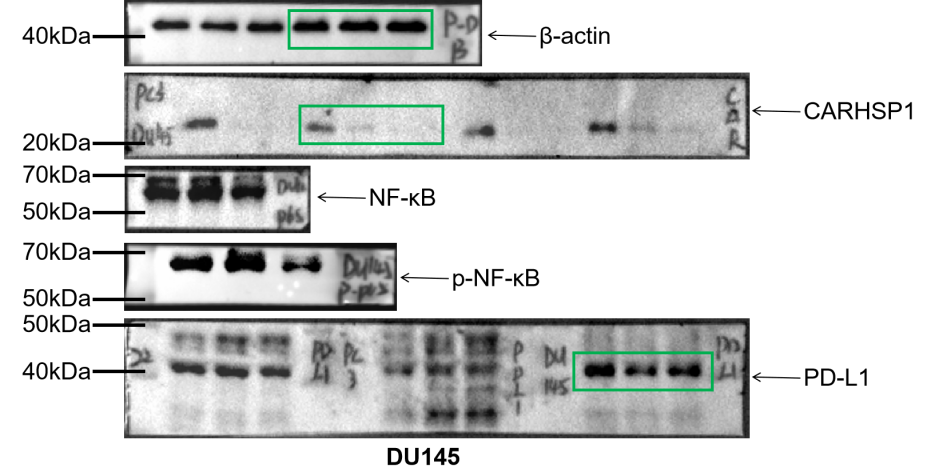

Supplement: Supplementary file 2 — Supplementary Material 2 [file 13578_2025_1371_MOESM2_ESM.docx]
